# Glycogen synthase 1 targeting reveals a metabolic vulnerability in triple-negative breast cancer

**DOI:** 10.1186/s13046-023-02715-z

**Published:** 2023-06-06

**Authors:** E. C. de Heer, C. E. Zois, E. Bridges, B. van der Vegt, H. Sheldon, W. A. Veldman, M. C. Zwager, T. van der Sluis, S. Haider, T. Morita, O. Baba, C. P. Schröder, S. de Jong, A. L. Harris, M. Jalving

**Affiliations:** 1 Department of Medical Oncology, University of Groningen, University Medical Center Groningen, PO Box 30.001, 9700 RB Groningen, The Netherlands; 2 Department of Oncology, Weatherall Institute of Molecular Medicine, University of Oxford, Hypoxia and Angiogenesis Group, Cancer Research UK Molecular Oncology Laboratories, Oxford, OX3 9DS UK; 3 Department of Radiotherapy and Oncology, School of Health, Democritus University of Thrace, Alexandroupolis, Greece; 4 Department of Oncology, MRC Weatherall Institute of Molecular Medicine, John Radcliffe Hospital, Molecular Oncology Laboratories, Oxford University, Oxford, OX3 9DS UK; 5 Department of Pathology and Medical Biology, University of Groningen, University Medical Center Groningen, Groningen, the Netherlands; 6 The Breast Cancer Now Toby Robins Research Centre, The Institute of Cancer Research, London, UK; 7 Tokushima University Graduate School, 3-18-15, Kuramoto-Cho, Tokushima, 770-8504 Japan; 8 Department of Medical Oncology, Antoni Van Leeuwenhoek-Netherlands Cancer Institute, Amsterdam, the Netherlands

**Keywords:** Glycogen synthase 1, Breast cancer, Glycogen, Mitochondria, Ki67

## Abstract

**Background:**

Hypoxia-induced glycogen turnover is implicated in cancer proliferation and therapy resistance. Triple-negative breast cancers (TNBCs), characterized by a hypoxic tumor microenvironment, respond poorly to therapy. We studied the expression of glycogen synthase 1 (*GYS1*), the key regulator of glycogenesis, and other glycogen-related enzymes in primary tumors of patients with breast cancer and evaluated the impact of *GYS1* downregulation in preclinical models.

**Methods:**

mRNA expression of *GYS1* and other glycogen-related enzymes in primary breast tumors and the correlation with patient survival were studied in the METABRIC dataset (*n* = 1904). Immunohistochemical staining of GYS1 and glycogen was performed on a tissue microarray of primary breast cancers (*n* = 337). In four breast cancer cell lines and a mouse xenograft model of triple-negative breast cancer, *GYS1* was downregulated using small-interfering or stably expressed short-hairpin RNAs to study the effect of downregulation on breast cancer cell proliferation, glycogen content and sensitivity to various metabolically targeted drugs.

**Results:**

High *GYS1* mRNA expression was associated with poor patient overall survival (HR 1.20, *P* = 0.009), especially in the TNBC subgroup (HR 1.52, *P* = 0.014). Immunohistochemical GYS1 expression in primary breast tumors was highest in TNBCs (median H-score 80, IQR 53–121) and other Ki67-high tumors (median H-score 85, IQR 57–124) (*P* < 0.0001). Knockdown of *GYS1* impaired proliferation of breast cancer cells, depleted glycogen stores and delayed growth of MDA-MB-231 xenografts. Knockdown of *GYS1* made breast cancer cells more vulnerable to inhibition of mitochondrial proteostasis.

**Conclusions:**

Our findings highlight GYS1 as potential therapeutic target in breast cancer, especially in TNBC and other highly proliferative subsets.

**Supplementary Information:**

The online version contains supplementary material available at 10.1186/s13046-023-02715-z.

## Background

Triple-negative breast cancers (TNBCs), representing15-20% of all breast cancers, lack expression of both hormone receptors and lack overexpression of human epidermal growth factor receptor 2 (HER2) [1]. TNBC is an aggressive subtype, characterized by rapid proliferation, a high metastatic capacity and rapid resistance to chemotherapy [2]. This biology and lack of anchors for targeted therapy result in a worse prognosis for these patients compared to patients with an estrogen receptor (ER) and/or HER2 + breast tumor [3].

**Fig. 1:**
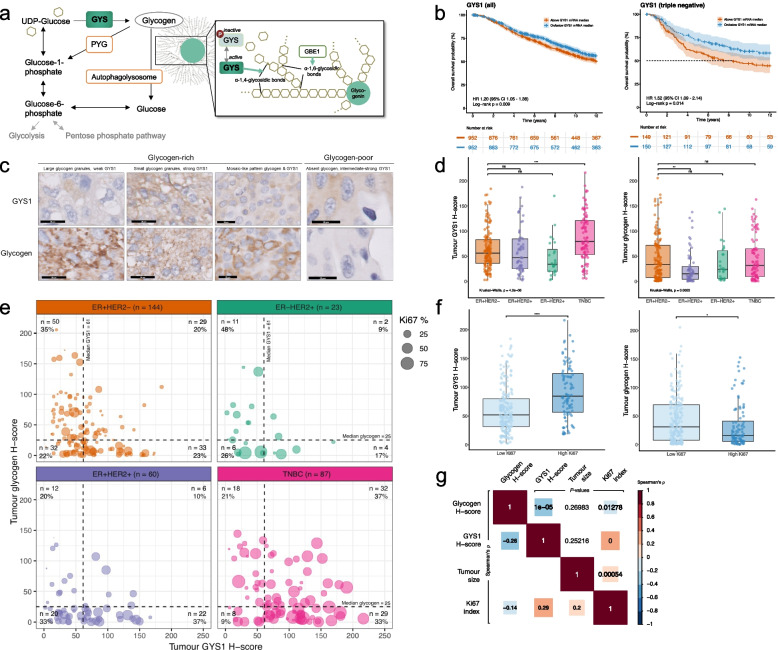
Expression patterns of GYS1 and glycogen in breast cancer patients. **a** Schematic overview of glycogen metabolism and the role of GYS1, which catalyzes formation of the α-1,4-glycosidic bonds between UDP-glucose molecules. **b** Overall survival of respectively all (left panel) and triple-negative (right panel) breast cancer patients from the METABRIC cohort (*n* = 1904) with respectively high or low *GYS1* mRNA expression in their primary tumor [22]. Curves are separated by the median *GYS1* expression. **c** Representative examples of differing staining patterns in glycogen-rich and glycogen-poor primary breast tumor areas. 60 × magnification, scalebar represents 20 μm. **d** Tukey boxplots of staining scores of all primary breast tumors by clinical subtype. Displayed are median, interquartile range (boxes) and 1.5 × interquartile range (whiskers). **e** Scatterplot of GYS1 (x-axis) and glycogen H-scores (y-axis) and Ki67 proliferation index (dot size) of primary breast tumors, stratified by subtype (color). **f** Tukey boxplots of staining scores of primary breast tumors, stratified by Ki67 proliferation index. Displayed are median, interquartile range (boxes) and 1.5 × interquartile range (whiskers). **g** Spearman correlations (lower left triangle) and corresponding *P-*values (upper right triangle) of staining scores with tumor size and Ki67 index. *CI* = *confidence interval; GBE* = *glycogen branching enzyme; GYS* = *glycogen synthase; HR* = *hazard ratio; PYG* = *glycogen phosphorylase; UDP* = *uridine diphosphate*

An hypoxic tumor microenvironment contributes to tumor progression, infiltration and therapy resistance, and is common in TNBCs [4, 5]. Essential metabolic pathways that are upregulated by hypoxia-induced factors are attractive as potential drug targets, especially since direct therapeutic targeting of tumor hypoxia is notoriously difficult [6]. Glycogen metabolism is one of the metabolic pathways that is upregulated in hypoxia. Glycogen contributes to the glucose supply required for glycolysis, the pentose phosphate pathway, and the tricarboxylic acid cycle [7]. Glycogen molecules consist of chains of glucose polymers that are attached to one central core protein glycogenin [8, 9]. Individual glucose molecules modified by uridine diphosphate (UDP) are covalently bound through α-1,4-glycosidic bonds by activated glycogen synthase (GYS) to form polymers [7, 8, 10] (Fig. 1a). GYS isoform 1 (*GYS1*), the fundamental regulator of glycogen synthesis in non-liver tissues [11], and glycogen breakdown enzymes (glycogen phosphorylases, *PYG*) are all HIF-regulated and upregulated in hypoxia [12–15]. Preclinically, cancer cells, including breast cancer cells, show elevated intracellular glycogen concentrations which are further increased during hypoxia [12–14, 16–19]. In addition, glycogen accumulation in breast cancer cells is induced by radiation and is associated with radioresistance [20, 21]. Metformin, which inhibits mitochondrial complex I, decreased intra-cellular glycogen levels and reversed radioresistance [20]. Collectively, these data suggest that cancer cell glycogen metabolism contributes to cell survival in stressful or nutrient-limiting conditions such as those induced by hypoxia, poor vascularization, high proliferation or therapy.

Glycogen metabolism may thus provide novel therapeutic targets in breast cancer. High tumor expression of glycogen-related enzymes is associated with worse patient outcomes in multiple other cancer types [23–27]. In human breast cancers, the expression of the key glycogen synthesis enzyme GYS1, its correlation with tumor glycogen levels and the functional consequences of *GYS1* downregulation are unknown. Therefore, we evaluated *GYS1* mRNA levels in publicly available breast cancer data and immunohistochemical expression of GYS1 and glycogen in primary human breast cancers. We subsequently investigated the impact of *GYS1* knockdown on proliferation, glycogen content and sensitivity to mitochondrial targeting in a panel of breast cancer cell lines and in a xenograft model, focusing on TNBC.

## Methods

### Patient mRNA data and tissue-microarray

Robust multiarray average (RMA)-normalized mRNA microarray expression data from the publicly available METABRIC dataset [22] and accompanying clinical data were downloaded on 7^th^ November 2021 from cBioPortal (https://www.cbioportal.org/study/summary?id=brca_metabric). Cases with missing *GYS1* expression data were excluded from all analyses.

For the patient tissue-microarray (TMA), tissue of primary breast tumors obtained during primary surgery was retrieved from patients who were treated in the University Medical Centre Groningen (UMCG) between 2006 – 2012. The tissue used is regarded as residual tissue., therefore need for informed consent was waived according to institutional and national guidelines. Clinical data were derived from the electronic patient file, pseudonymized and stored on a secured server following local guidelines.

### Immunohistochemistry

In the patient TMA, three cores containing viable tumor tissue were included from every resection specimen. In addition, cores from the following tissues were included in duplicate or triplicate on each TMA: non-malignant mamma tissue, liver, kidney, gall bladder, colon, placenta. Paraffin sections of 3 µM thickness were applied to APES-coated slides (Starfrost, Braunschweig, Germany). Following heat-induced antigen retrieval and blockage of endogenous peroxidase (500 µl 30% H_2_O_2_ / 50 ml PBS for 30 min), slides were incubated with the indicated primary antibodies against GYS1, carbonic anhydrase 9 (CA9), glycogen [28, 29], cytokeratin 8/18 and Ki67 (see Suppl. Table 2 for buffer and antibody specifications). Staining was visualized after incubation with biotinylated or peroxidase-bound secondary antibodies using streptavidin–biotin/horseradish peroxidase complex (Dako, USA, 1:100) and 3,3′-diaminobenzidine (Sigma Aldrich). For periodic acid-Schiff (PAS) staining, slides with and without pretreatment with 0.8% a-amylase type VI-B (30 min, 37 °C, Sigma Aldrich) were incubated with 1% periodic acid (10 min, VWR chemicals) followed by Schiff’s reagent (15 min, Merck). Haematoxylin (Klinipath) was used as counterstain.

Stained slides were analyzed using Visiopharm software (Visiopharm, Hørsholm, Denmark). Percentage of tumor area per core was determined using an in-house tumor detection app, and manually adjusted if necessary based on cytokeratin 8/18 staining of the same core. Cores with < 5% tumor tissue in the region-of-interest, < 10% tumor tissue of the total core tissue and/or with major artefacts resulting in no evaluable tumor tissue were excluded from further analysis. Scores for GYS1, glycogen and Ki67 were derived by an in-house app that delineates areas of weak (intensity 1), intermediate (2) and strong (3) cytoplasmic staining using predefined constant thresholds (see representative examples in Suppl. Figure 3c). Minimum and maximum thresholds, respectively, were set based on positive and negative controls. As positive controls for GYS1 and glycogen, respectively, areas with striated muscle and liver cores were designated as ‘strong’. The final score for each staining was determined by multiplying the percentage of tumor area with a certain intensity, similar to the H-score, resulting in a final score between 0–300. Tumor Ki67 was scored using a previously validated Visiopharm app, and proliferation index (%) was determined as (positive nuclei/total nuclei)*100[30]. For GYS1 and glycogen scores, the first 60 consecutive cores were manually assessed and scored by a breast cancer pathologist (BvdV) to verify correct scoring by the app (data not shown). Additionally, glycogen antibody staining scores were qualitatively compared to PAS ± diastase staining of the same core.

### Cell culture and transfection with siRNA and shRNA

Breast cancer cell lines MDA-MB-231, MCF-7, HCC-1806, SUM-159PT, BT474, SKBR3, MDA-MB-453, T47D, MDA-MB-436, CAL51 and MDA-MB-468 and the glioblastoma cell line U87 MG were obtained from ATCC. All cell lines were STR-profiled and regularly tested to be mycoplasma-free. Cells were maintained in a humidified atmosphere at 37 °C and 5% CO_2_ in Dulbecco’s Modified Eagle Medium (DMEM) containing 25 mM glucose (#41,966–029, Gibco), supplemented with 10% fetal calf or bovine serum (Life Technologies, Waltham, MA, USA). Cell lines were passaged twice a week up to a maximum of 40 passages.

For acute *GYS1* knockdown experiments, cells were transfected with pool or single sequence small interfering RNAs targeted against *GYS1* (Invitrogen) or a scrambled control (Dharmacon) (final concentration 20 nM, sequences in Suppl. Table 2) in Lipofectamine RNAiMAX (ThermoFisher) and OptiMEM (Gibco) according to manufacturer’s instructions. MDA-MB-231 and MDA-MB-231-Brain cell lines with stable *GYS1* knockdown were constructed by lentiviral transduction with MISSION® short hairpin RNA (shRNA) transduction particles (Sigma-Aldrich) targeted against *GYS1* or a non-targeting shRNA sequence (Suppl. Table 2). Cells were transduced with a multiplicity of infection of 3, in the presence of 6 mg/ml Polybrene (Sigma). Cells expressing the shRNA were selected in puromycin (Sigma)-containing medium (1 mg/ml). Subsequently, sh*GYS1* cell lines were maintained under selective pressure in 25 mM DMEM + 10% FCS supplemented with 0.01% puromycin.

### Proliferation and viability assays

In short-term siRNA proliferation assays, 1 × 10^5^ cells were seeded in 3 ml 5.6 mM glucose DMEM + 10% fetal calf serum (FCS) in 6-wells plates. Medium was refreshed daily to mimic constant physiological glucose levels and at day 5, cells were harvested by trypsinization, resuspended and counted. For hypoxic conditions, a Ruskinn hypoxic workstation was used for 0.1% O_2_ conditions and a Sanyo CO_2_ incubator for 1% O_2_ conditions. For spheroid formation, MDA-MB-231 or HCC1806 cells were transfected with si*GYS1* or scrambled control and harvested after 24 h by trypsinization. Cells were resuspended in complete DMEM containing 2.5% matrigel (Corning #354,230), seeded in 96-wells ultra-low attachment round bottom plates (Corning #7007) at 5.000 cells/well and plates were centrifuged to initiate spheroid formation (10 min, 1000 g, 4C). On day 3 after seeding, 100μl of fresh medium was added. Every other day, pictures of every well were taken (EVOS Cell Imaging system, ThermoFisher). For clonogenic assays, MDA-MB-231 or HCC1806 cells were seeded 1 day after transfection with siCtrl or si*GYS1.* They were seeded in 5.6 mM glucose complete DMEM + 3% agarose in 6-wells Petri dishes (2000 cells/well) that were precoated with complete medium + 3% agar.

For short-term inhibitor experiments, cells were seeded in 96-wells plates (2.000 cells/well, 10 mM glucose complete DMEM), left to adhere overnight and treated with for 96 h with increasing concentrations of NBS-037 (Novintum Bioscience, London, UK) [31], gamitrinib-triphenylphosphonium (GTPP, kind gift from D. Altieri) or phenformin (Sigma Aldrich, P7045). 3-(4,5-dimethylthiazol-2-yl)-2,5 diphenyltetrazolium methyl thiazolyl tetrazolium (MTT, Sigma Aldrich M2128) was added after 96 h (final concentration: 0.5 mg/ml) and cells were incubated for four additional h. After centrifuging (15 min, 900 rpm) and careful medium aspiration, 200 μl DMSO (Sigma) was added to dissolve formazan crystals and absorbance was measured at 520 nm with an iMark absorbance reader (BioRad). In several experiments, duplicate plates were fixed and stained with crystal violet to verify MTT results by a non-metabolic method (data not shown).

For long-term NBS-037 experiments, cells were seeded in 48-wells plates (3.000 cells/well) in complete DMEM containing resp. 1 mM or 5.6 mM glucose and left to adhere overnight. Half of the medium was aspirated every other day and supplemented with fresh complete medium containing either DMSO control or NBS-037 (first week) or only fresh complete medium (second week) to ensure relatively stable glucose levels. Well confluency was measured every 3 h using IncuCyte™ ZOOM (Essen BioScience, Essen, Germany).

### Glycogen content

Cellular glycogen content was measured colorimetrically using the Glycogen Colorimetric/Fluorometric Assay Kit (K646-100, BioVision Inc., Milpitas, Santa Clara, CA, USA). Cells were plated in 10-cm^2^ dishes (7 × 10^5^ cells/dish), left to seed overnight and placed in normoxia or 1% hypoxia. After 48 h, cells were washed twice with ice-cold PBS, scraped, and cell pellets were collected and snap-frozen in liquid nitrogen. According to manufacturer’s instructions, cell pellets were homogenized in 200 μl sterile H_2_O and homogenized using 25G syringes. Homogenates were boiled for 10 min to inactivate enzymes and spun down, and the supernatant was assayed for glycogen content according to manufacturer’s instructions, including sample readings without amyloglucosidase addition to measure the glucose background. Glycogen content was normalized to protein content determined by Bradford assay.

### Western blotting

Cell lysates were obtained by scraping cells in RIPA buffer (Thermo Scientific, Waltham, MA, USA), supplemented with respectively 1:100 protease and phosphatase inhibitor (ThermoFisher). Protein concentration was estimated using Bradford assays. Per sample, 25 μg protein was loaded and separated by SDS-PAGE. Separated proteins were blotted onto PVDF membranes (Millipore, Burlington, MA, USA). Membranes were blocked in 5% skim milk (Sigma, St. Lois, MO, USA) in 0.05% Tween20-TBS and incubated overnight at 4 °C with primary antibodies (see Suppl. File 2 for specifications). After washing, membranes were incubated with appropriate HRP-conjugated secondary antibodies (DAKO, Santa Clara, CA, USA). Proteins were visualized using LumiLight (Roche, Basel, Switzerland) on a ChemiDoc MP imaging system (BioRad, USA).

### Xenograft experiments

Female BALB/c SCID mice of 5–6 weeks old (weight 20 gr) were used for this study. Under anesthesia, of 10^6^ cells of MDA-MB-231 transfected with shCtrl or sh*GYS1*, respectively, in 50 μl Serum Free Media mixed with Matrigel (1:1) of were subcutaneously injected into the mammary fat pad of each mouse. Tumor growth was monitored three times per week by measuring the length (L), width (W), and height (H) of each tumor using calipers. Volumes were calculated using the equation (LxWxH)^1/3^ (mm^3^). The experiment was terminated at day 58 when the first tumor reached the size of 12 mm. Mice were killed by cervical dislocation and tumors were resected, formalin-fixed and embedded in paraffin or snap-frozen for RNA extraction, respectively. 3–5 μm thick slices were stained by IHC for GYS1, Ki67 and by PAS ± diastase, and GYS1 and Ki67 were analyzed by Visiopharm with the same quantification apps as used in the patient TMA.

### Data analysis

Spearman correlation coefficients were used for correlations between mRNA expression levels. Kaplan–Meier survival curves were separated by median *GYS1* expression of resp. the entire dataset or the indicated clinical subtypes and compared by log-rank tests. Non-parametric Kruskal–Wallis tests followed by post-hoc Dunn tests, or proportion Z-tests with Holm’s correction for multiple testing were used to assess differences in *GYS1* and glycogen expression between clinicopathological characteristics. Spearman’s correlation coefficients were used for correlations. Prior to unsupervised hierarchical clustering with (1-Spearman correlation) as distance measure and Ward.D2 as linkage method, *GYS1*, glycogen and Ki67 scores were square-root transformed to approach normal distributions followed by z-score normalization. Experimental data are derived from at least three independent experiments and displayed as mean or median ± SD unless mentioned otherwise. Conditions were compared using unpaired t-tests with Holm-Sidak’s correction for multiple comparisons and/or ANOVA. Spheroid sizes were determined from microscope images using the open-source MATLAB software package SpheroidSizer. All statistical analyses and data visualization were performed in GraphPad Prism (v.8.4) and R (v.4.0.0 and Rstudio v.1.2.5042, packages: ggplot2 v.3.3.5, survival v.3.2, corrplot v.0.90, ComplexHeatmap v2.11.1, rstatix v.0.7.0). A two-sided, adjusted *P*-value < 0.05 was considered statistically significant.

## Results

### *GYS1* mRNA expression in primary breast cancers is associated with overall survival in triple-negative breast cancer patients

We first explored the associations between patient survival and breast tumor mRNA expression of enzymes involved in glycogen metabolism in the publicly available METABRIC dataset. METABRIC contains well-annotated mRNA microarray expression data from the primary breast tumors from two patient cohorts with long-term follow-up (*n* = 1904) [22]. Of the enzymes involved in glycogen synthesis, GYS1 and glycogen branching enzyme 1 (GBE1) were ubiquitously expressed whereas the isoform GYS2 was restricted to the liver and had low to no expression in non-liver tumors [32]. Only high *GYS1* mRNA expression was associated with worse patient overall survival in the entire breast cancer cohort (Fig. 1b, hazard ratio [HR] 1.20 (95% confidence interval [CI] 1.05–1.38), log-rank *P* = 0.009; Suppl. Figure 1a). None of the glycogen phosphorylase isoforms correlated with survival in this dataset. When the subtypes were analyzed separately, the worse overall survival for *GYS1*-high patients was observed in TNBC but not in the other subtypes (Fig. 1b, Suppl. Figure 1b-c; HR 1.52 [95% CI 1.09–2.14], *P* = 0.014). This association with worse overall survival for patients with *GYS1*-high TNBC remained present when correcting for tumor size, grade and number of positive lymph nodes by using the Nottingham Prognostic Index, a clinically validated prognostic index [33] (Suppl. Figure 1d, HR 1.41 [95% CI 1.00–1.98], Wald’s* P* = 0.049). Correlations between *GYS1* levels and mRNA levels of other key enzymes of glycogen metabolism, the well-established hypoxic marker carbonic anhydrase 9 (*CA9*) and the proliferation marker *MKI67* were poor to moderate, ranging from *p* = -0.26 for *GBE1* (*P* < 0.001) to 0.26 for *MKI67*; Suppl. Figure 2a, *P* < 0.001). Absolute *GYS1* mRNA levels were highest in the ER-/HER2 + subtype and in grade 3 tumors (Suppl. Figure 2b). There were no differences in *GYS1* mRNA expression between tumors from different stages.

### Immunohistochemical GYS1 expression is highest in triple-negative primary breast tumors

High mRNA expression of *GYS1,* but not of the other glycogen synthesis or degradation enzymes, was associated with worse survival in breast cancer patients, especially in patients with TNBC. Therefore, we focused on GYS1 protein expression and its association with glycogen content and clinicopathological characteristics in a tissue microarray (TMA) constructed with tissue from human breast tumors obtained during primary surgery (Suppl. Figure 3a). In total, 337 tumors were included, of which 217 were ER + (64%), 95 HER2 + (28%) and 93 triple-negative (28%) (see Suppl. Table 1 for patient and tumor characteristics). GYS1- and glycogen-positivity were observed in the cell cytoplasm only. GYS1 staining was diffuse in the cytoplasm whereas glycogen staining showed a clear granular morphology (Fig. 1c). Notably, high glycogen was observed in both GYS1-negative and GYS1-positive areas (Fig. 1c). The glycogen patterns were confirmed by PAS ± diastase staining of the same area (Suppl. Figure 3b, c). Glycogen- and GYS1-positive areas did not consistently coincide with CA9-positivity in the same core, and multiple tumors with strong GYS1 and/or CA9 expression lacked glycogen (Suppl. Figure 3b).

GYS1 scores were highest in the TNBC subtype (median H-score 80, IQR 53–121), compared to ER + HER2- (used as reference group; median 56, IQR 36–83), ER + HER2 + (median 47, IQR 25–85) and ER-HER2 + (median 34, IQR 20–64)(*P* = 0.00054, Fig. 1d). Glycogen scores were lowest in ER + HER2 + tumors (median H-score 15, IQR 4–30) vs. ER + HER2- (median 34, IQR 8–72), ER-HER2 + (median 24, IQR 6–61) and TNBC tumors (median 32, IQR 9–65) (*P* = 0.0064, Fig. 1d). When dividing tumors into quadrants defined by the median GYS1 and glycogen scores, the largest subset of both ER + HER2- and ER-HER2 + tumors was GYS1-low/glycogen-high (35% and 48%, respectively; Fig. 1e). Within ER + HER2 + tumors, the most prevalent combination was GYS1-high/glycogen-low (37%) and within TNBCs, this was GYS1-high/glycogen-high (37%; Fig. 1e).

### Ki67-high primary breast tumors have high GYS1 protein expression and low glycogen

Primary breast tumors with a high Ki67 proliferation index, using a cut-off of 20% according to ESMO Early breast cancer guidelines [34], had higher GYS1 expression than Ki67-low tumors (median H-score 85, IQR 57–124 vs. 52, IQR 32–80; *P* < 0.0001; Fig. 1f, Suppl. Figure 3d). GYS1 did not differ between tumor grades (Suppl. Figure 3d). Glycogen levels were lowest in Ki67-high tumors (median H-score 16, IQR 6–41 vs. 31, IQR 8–70; *P* = 0.014) and grade 3 tumors (median H-score 19, IQR 5–55; *P* = 0.021; Fig. 1f, Suppl. Figure 3d). Overall, higher GYS1 was moderately correlated with higher Ki67 (Spearman’s p = 0.29, *P* < 0.0001) and lower glycogen scores (Fig. 1g; *p* = -0.26, *P* < 0.0001). Tumor size did not correlate with GYS1 or glycogen levels. To distinguish potential subgroups among breast tumors based on GYS1, glycogen and Ki67 scores, we performed unsupervised hierarchical clustering. Of the three major clusters, cluster one contained predominantly Ki67-high, grade 3 tumors with relatively high GYS1 and low glycogen levels; cluster two contained Ki67-low tumors with lower GYS1 and higher glycogen expression than cluster one; and tumors in cluster three had relatively high glycogen, mixed Ki67-levels and the lowest GYS1 levels of all clusters (Suppl. Figure 3e). Exploratory overall survival analysis revealed no differences in survival when patients were stratified by GYS1 or glycogen scores (Suppl. Figure 3f). In short, GYS1 is expressed in the majority of primary breast tumors, with the highest levels in TNBCs and other Ki67-high tumors.

### GYS1 is heterogeneously expressed in breast cancer cell lines and knockdown impairs proliferation in normoxia and hypoxia

**Fig. 2:**
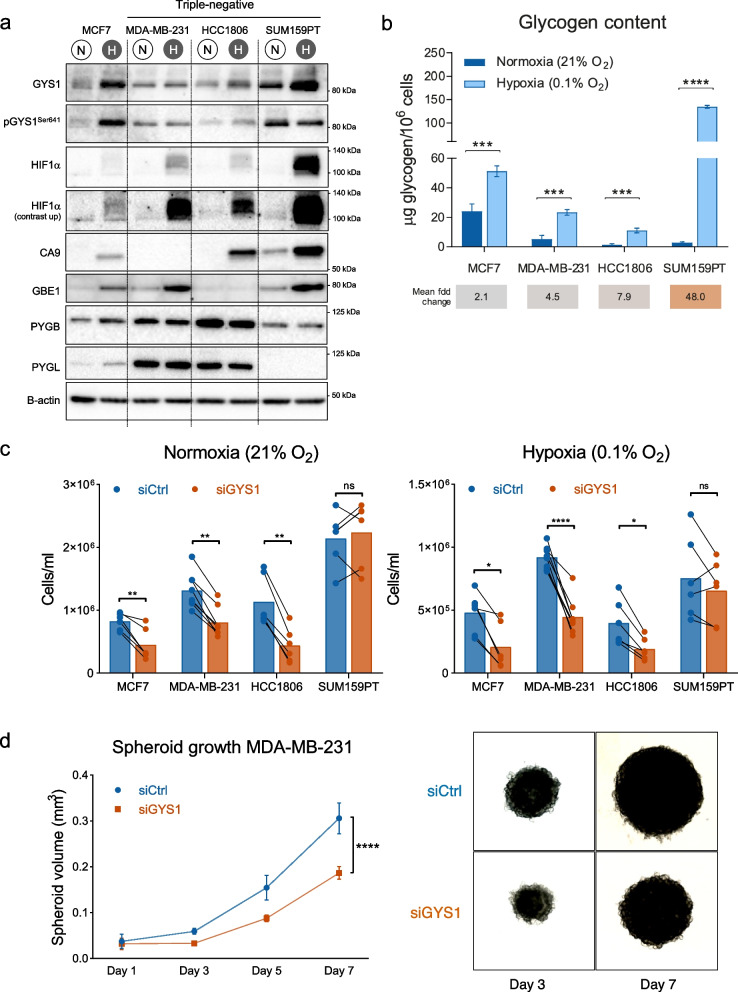
GYS1 and glycogen are increased in hypoxia and acute GYS1 knockdown impairs breast cancer proliferation. **a** Protein expression of total GYS1, pGYS1(Ser641) (inactive form), the hypoxic markers HIF1 α and CA9, and other key enzymes in glycogen metabolism GBE1, PYGB and PYGL. Cells were cultured for 24 h in normoxia (N) or 1% hypoxia (H). **b** Glycogen levels after 24 h normoxia or 1% hypoxia, normalized by cell number (mean ± SD). **c** Breast cancer cell number after 5 days with siRNA-mediated GYS1 knockdown or scrambled control in normoxia (left) and 0.1% hypoxia (right) **d** Spheroid growth of MDA-MB-231 cells transfected with siRNA-mediated GYS1 knockdown or scrambled control. *CA* = *carbonic anhydrase; GBE* = *glycogen branching enzyme; GYS* = *glycogen synthase; HIF* = *hypoxia-inducible factor; PYGB* = *brain glycogen phosphorylase; PYGL* = *liver glycogen phosphorylase; ns* = not significant, *P < 0.05, **P < 0.01, ***P < 0.005, ****P < *0.001*

Next, we studied GYS1 expression and impact of GYS1 downregulation in a panel of breast cancer cell lines. All cell lines expressed GYS1 protein (Fig. 2a, Suppl. Figure 4a). *GYS1* knock-down inhibited growth of most breast cancer cell lines (Suppl. Figure 4b). Based on the association of tumor *GYS1* mRNA expression with overall survival in patients with TNBC specifically and their high GYS1 protein expression, we selected three TNBC cell lines (HCC1806, MDA-MB-231, SUM159PT) and one ER + cell line (MCF7) for further analyses. GYS1 expression increased after 24 h hypoxia, with the greatest induction in MCF7 and SUM159PT cells and only marginal induction in MDA-MB-231 cells. Hypoxia increased phosphorylated GYS1 (pGYS1) in MCF7 but not in SUM159PT cells, suggesting the greatest activity of glycogen synthesis of SUM159PT cells in response to hypoxia. Hypoxic induction was especially pronounced for GBE1 and CA9, unlike PYGL and PYGB (Fig. 2a).

The glycogen content was increased in all cell lines after 24 h hypoxia (0.1% O_2_) (Fig. 2b). Hypoxic glycogen induction was more pronounced in TNBC cell lines compared to the ER-positive cell line MCF-7, with mean fold changes in hypoxia ranging from 2.1 for MCF-7 to 48 for SUM159PT. In normoxia and hypoxia, si*GYS1* decreased 2D cell proliferation after 5 days of most cell lines, except for SUM159PT (Fig. 2c, Suppl. Figure 4b). There were no changes in degree of apoptosis (data not shown). Clonogenic potential of MDA-MB-231 and HCC1806 were also reduced in *GYS1* knockdown cells (Suppl. Figure 5b and 6a). To mimic 3D tumors with a hypoxic core, MDA-MB-231 spheroids ± si*GYS1* were generated. After 7 days, spheroid size was smaller in *GYS1* knockdown cells compared to controls (Fig. 2d), but the Ki67 proliferation index did not differ (data not shown). HCC1806 spheroids ± si*GYS1* were also generated. These spheroids were too small to quantify, but Ki67 proliferation index was lower in GYS1 knockdown spheroids (Suppl. Figure 6b). Taken together, our results show that acute downregulation of *GYS1* impairs breast cancer proliferation in vitro in 2D and 3D in both hypoxia and normoxia.

### GYS1 knockdown depletes glycogen and impairs MDA-MB-231 xenograft growth

**Fig. 3:**
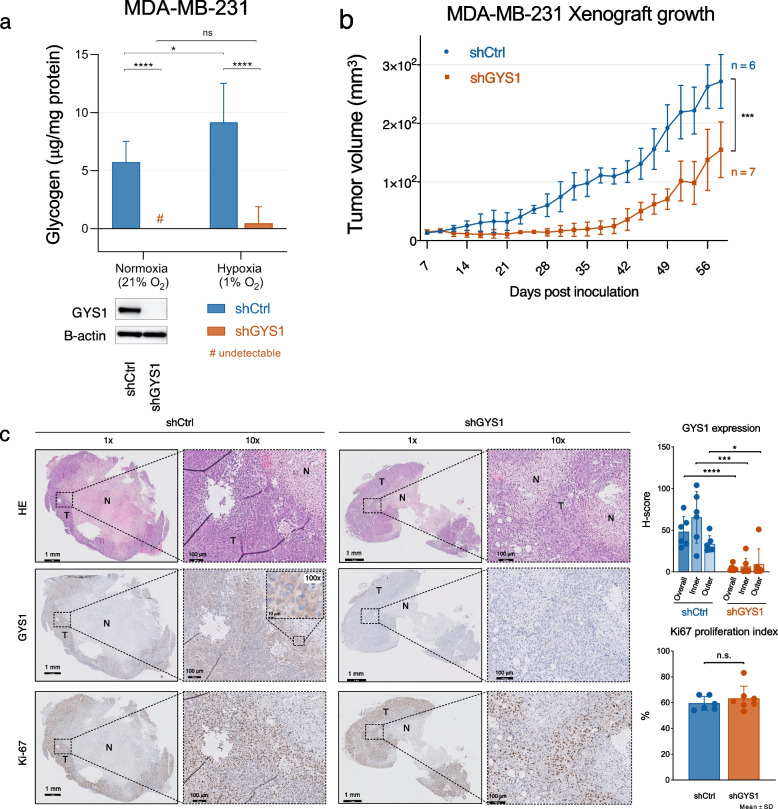
Stable GYS1 knockdown depletes glycogen stores and diminishes growth of TNBC xenografts (**a**) Glycogen levels of MDA-MB-231 cells ± shGYS1 after 48 h growth in 5.6 mM glucose DMEM in normoxia or 1% hypoxia, normalized by protein levels (mean ± SD). **b** Growth of subcutaneous MDA-MB-231 tumors with shGYS1 (*n* = 7 mice) or scrambled control (*n* = 6; one xenograft tumor did not engraft and was omitted from analyses) (mean ± SD). **c** Representative examples of HE and immunohistochemical GYS1 and Ki67 staining in shCtrl or shGYS1 xenografts. The 100 × magnification inset shows that GYS1 was exclusively detected in the cytoplasm. GYS1 expression was quantified in the complete vital tumor area (overall) and in regions-of-interest adjacent to the necrotic tumor core (inner) or outer tumor border (outer) resp. Scalebars represent 1 mm for 1 × magnification, 100 μm for 10 × and 10 μm for 100x. *ns* = not significant, *P < 0.05, **P < 0.01, ***P < 0.005, ****P < *0.001. N* = *necrosis, T* = *vital tumor*

To evaluate tumor growth upon long-term vs. acute ***GYS1***-knockdown and in an in vivo setting, we constructed a short-hairpin ***GYS1***-knockdown MDA-MB-231 cell line. MDA-MB-231 sh***GYS1*** cells were glycogen-depleted compared to shCtrl cells, while basal intracellular glucose levels did not differ between shCtrl and sh***GYS1*** cells (Fig. 3a, Suppl. Figure 5c). MDA-MB-231 xenograft growth was impaired with ***GYS1***-knockdown (Fig. 3b), especially in the first six weeks after engraftment, after which sh***GYS1*** xenografts regained a similar growth rate to controls. Tumors harvested at termination of the experiment (day 58) were stained for GYS1, Ki67 and with PAS ± diastase staining. Cytoplasmic GYS1 staining was present in all shCtrl xenografts, with higher intensity in areas adjacent to the necrotic tumor core compared to the outer tumor border (Fig. 3c). GYS1 staining was absent, and strongly reduced *GYS1* mRNA levels confirmed knockdown, in all but one of the sh***GYS1*** xenografts (Suppl. Figure 7a, b). In this single xenograft #7, half of the tumor showed re-expression of ***GYS1*** mRNA and protein whereas the other half was negative for ***GYS1***. Ki67 proliferation index did not differ between shCtrl vs. sh***GYS1*** tumors, reflecting the equal proliferation at towards the end of the experiment (Fig. 3c). PAS-staining showed little to no positivity and CA9-positive cells were scarce in both groups, corresponding to a low glycogen content and the absence of CA9 in the Western blot analysis of MDA-MB-231 cells in vitro (Fig. 2a, Suppl. Figure 7c). Thus, ***GYS1*** downregulation caused intracellular glycogen depletion and delayed TNBC xenograft growth.

### Stable GYS1 knockdown sensitizes triple-negative breast cancer cells to mitochondrial inhibition

**Fig. 4:**
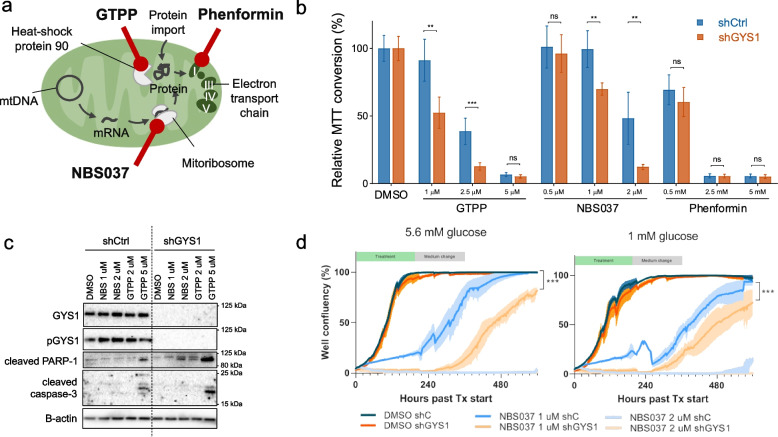
shGYS1 breast cancer cells are more sensitive to inhibition of mitochondrial protein proteostasis (**a**) Schematic overview of the targets of respectively gamitrinib-triphenylphosphonium (GTPP) (i.e., targeting heat-shock protein 90), NBS-037 (the mitoribosome) and phenformin (mitochondrial complex I). **b** 3-(4,5-dimethylthiazol-2-yl)-2,5-diphenyltetrazolium bromide (MTT) conversion, normalized to DMSO-treated cells, of MDA-MB-231 cells ± shGYS1 or shCtrl after 5 days of treatment with GTPP, NBS037 or phenformin (mean ± SD). **c** Western blot of apoptotic markers after 48 h of treatment with NBS037 or GTPP. **d** Well confluency of MDA-MB-231-shCtrl and -shGYS1 cells treated with different concentrations of NBS-037, cultured in resp. 1 mM or 5.6 mM glucose complete DMEM, measured by Incucyte every 3 h. After one week of drug exposure with refreshment of dose every other day, medium was changed every other day during week two to maintain constant glucose concentrations, after which cells were left to grow out. Lines represent mean and shaded areas 95% CI. *Peak at 240 h in 1 mM DMEM condition caused by a plate lid artefact. *ns* = not significant, *P < 0.05, **P < 0.01, ***P < *0.005*

After an initial delay, the eventual growth rate of sh*GYS1* xenografts matched that of shCtrls, which is in line with the observation that monolayer growth of sh*GYS1* cells did not differ from controls (Suppl. Figure 5d). This suggests that, in contrast to *GYS1* knockdown in an acute setting, cells adapt to chronic *GYS1*-knockdown without re-expression of *GYS1*. Blocking utilization of glycogen is known to increase dependency on mitochondrial respiration [13]. Moreover, patients with glycogen storage disease type 0b caused by congenital *GYS1* deficiency show increased mitochondrial proliferation and mitochondrial abnormalities in their muscle biopsies [35, 36]. We hypothesized that the adaptations induced by stable *GYS1* knockdown might convey sensitivity to mitochondrial targeting. We, therefore, investigated whether *GYS1*-knockdown cells were more sensitive to inhibitors of respectively mitochondrial complex I (phenformin), mitochondrial protein translation (compound NBS037, described in [31]) and the mitochondrial chaperone HSP-90 (gamitrinib-triphenylphosphonium, GTPP) (Fig. 4a). Sh*GYS1* cells were more sensitive to apoptosis inflicted by NBS037 and GTPP but not phenformin in short-term assays for up to five days (Fig. 4b, c). To evaluate long-term viability in a more clinical setting of long-term on–off drug schedules, sh*GYS1* cells were treated with NBS037 in fresh medium every other day for one week followed by a week of medium refreshment only every other day. Also in this long-term setting, *GYS1* knockdown sensitized MDA-MB-231 cells to NBS037 treatment (Fig. 4d). Of note, after three weeks of treatment with 2 $\upmu$ M NBS037, regrowth of shCtrl cells was observed but not of sh*GYS1* cells. These results imply that long-term inhibition of glycogen synthesis confers sensitivity to drugs that target mitochondrial proteostasis.

## Discussion

We demonstrate that the key glycogen synthesis enzyme GYS1 is expressed in most primary breast tumors, especially in triple-negative and Ki67-high tumors, and that knockdown of GYS1 impairs breast cancer proliferation in vitro and in vivo and enhances sensitivity to targeting of mitochondrial protein homeostasis. These observations support GYS1 as a potential therapeutic target, especially for patients with triple-negative and Ki67-high breast cancers.

Glycogen granules and GYS1 were detected across all breast cancer subtypes and grades. Previous case studies on glycogen metabolism in human breast tumors focused on the rare subtype of glycogen-rich clear cell carcinomas (GRCC) and reported an incidence of GRCC of only 0.01–2.8% [37–41]. These studies did not consistently describe GRCC definitions and/or glycogen detection methods, whereas we here used a sensitive glycogen antibody combined with semi-automatic quantification [28, 29]. This can explain the higher prevalence of glycogen-positive tumors in our study. GYS1 protein expression in breast cancer has not been previously described. The presence of the high GYS1/low glycogen pattern specifically suggest that a high glycogen turnover is related to the high proliferation rate of these aggressive subsets. High GYS1/low glycogen tumors were specifically observed in TNBCs and other Ki67-high tumors, which were captured in cluster one of an unsupervised cluster analysis. This could suggest that GYS1-driven glycogen stores are depleted to feed these aggressive rapidly growing tumors, in contrast to cluster two and three that contained tumors with high glycogen stores, variable GYS1 levels and lower proliferation rates. The inhibitory phosphorylation status of tumors with high GYS1 in those latter clusters are of interest for further evaluation. ER-positivity did not clearly explain GYS1 expression levels, although recent studies in other cell types, e.g., endometrial adenocarcinoma cells and rat astrocytes, demonstrated that estradiol stimulates GYS1 expression [42, 43]. Possibly, circulating estrogen levels are too low to influence glycogen synthesis in humans, especially since more than half of our study population was peri- or post-menopausal.

It is likely that local differences in nutrient availability and oxygen levels are important in the regulation of *GYS1* expression, GYS1 phosphorylation, i.e. inactivation, and its catalytic activity [44]. Here, we showed that expression of GYS1 protein and glycogen levels were indeed induced by hypoxia in all the breast cancer cell lines*.* Hypoxic GYS1 protein induction was lowest in the MDA-MB-231 cell line, in line with previous studies [19, 45]. Our in vivo data suggest a similar effect of hypoxia, since GYS1 protein expression in control xenografts was highest adjacent to the necrotic tumor core. The role of glycogen as an essential back-up fuel in stress conditions was previously shown [13, 14, 45]. Hypoxic preconditioning to induce glycogen stores increased the viability of breast cancer cells and hepatoma cells when the cells were subsequently cultured in glucose-depleted medium [12, 18]. However, we found that both *GYS1* mRNA and protein expression were only moderately correlated with expression of the hypoxia marker CA9, and *GYS1* expression was not limited to perinecrotic areas. Moreover, acute *GYS1* knockdown reduced cell proliferation of breast cancer cells also in normoxia and with glucose levels in the physiological range. This supports an essential role for glycogen synthesis and flux beyond that of simply a reserve energy pool only utilized during challenging conditions [7, 46]. Such a physiological function is seen in non-malignant brain cells and non-small cell lung cancer cells, where glycogen is involved in cell survival, protein glycosylation and epigenetic regulation by providing nuclear pyruvate for histone acetylation [47–49]. In neurons, glycogen-derived lactate supports neurotransmission of neighboring cells [50]. It is tempting to speculate that a similar glycogen-shunt between specific subsets of tumor cells with each other or surrounding cells from the tumor microenvironment could underlie the observed mosaic-like pattern of GYS1 and glycogen IHC expression in primary breast tumors.

In the short-term setting, knockdown of *GYS1* resulted in growth inhibition, but not in increased cell-death. The phenotype of growth inhibition was eventually lost in the longer-term in vitro and in vivo experiments. This phenomenon could not be explained by *GYS1* re-expression. This is in line with a previous study reporting that stable *GYS1* knock-out did not impair the growth of glycogen positive renal clear cell (RCC) cancer cells [51]. ^13^C-isotope flux analyses in RCC cells revealed that mitochondrial one-carbon metabolism is fueled by glycogen-derived glucose [51]. In addition, glycogen-debranching enzyme loss in bladder cancer increased reliance on interconversion of the one-carbon metabolites glycine and serine [27, 52]. These observations suggest that long-term *GYS1* downregulation may induce mitochondrial adaptations to overcome decreased flux through glycogen. Here, we actually found that sh*GYS1* breast cancer cells were more sensitive to mitochondrial proteostasis inhibitors but not to the mitochondrial complex I inhibitor phenformin. These findings may be applicable to other tumor types as well, as our results with U87 MG glioblastoma cell line model indicate (Suppl. Figure 6c). Taken together these results suggest that cancer cells that are deficient in glycogen synthesis may be especially vulnerable to mitochondrial targeting.

We used well-characterized cohorts of patients with breast cancer to study glycogen and *GYS1* mRNA and protein levels. Nevertheless, the retrospective nature of these patient cohorts and the cohort used for the immunohistochemical analyses is a limitation of our study. In addition, cohort used for IHC was underpowered to detect survival differences in the range of the hazard ratios as found in the METABRIC mRNA data. Furthermore, not all cell lines responded to *GYS1* inhibition, such as SUM159PT despite having high *GYS1* expression and glycogen stores. We were not able to pinpoint a specific determinant of sensitivity to *GYS1* knockdown. Other components contributing to glycogen synthesis and utilization (e.g., activity of GBE1, glycogenin and phosphorylases) are likely to be involved in determining sensitivity. Glycogen particle structure also influences the ability to release and utilize glucose from glycogen chains as demonstrated in glycogen storage diseases models [53–55]. Combining protein expression data from tumor immunohistochemistry, ^13^C-isotope flux analyses and structural particle measurements by Raman spectroscopy will help to elucidate the kinetics of glycogen metabolism in patients. These insights—along with a deeper mechanistic understanding of the long-term adaptations to *GYS1* knockdown and induced sensitivity to mitochondrial inhibitors—will help to select breast tumors that are most susceptible to therapeutic targeting of glycogen metabolism.

Multiple potent inhibitors of glycogen phosphorylases have been developed [56–59]. However, knockdown of glycogen phosphorylase in cancer cells induces different phenotypes dependent on the targeted isoform. Early clinical trials that evaluated these inhibitors have not published results or were hampered by toxicity, thereby stalling clinical development [13, 19, 45, 56, 59]. GYS1 constitutes an attractive alternative target in glycogen metabolism, due to its centrale role in glycogen synthesis. Inhibition of glycogen synthesis is currently being as a therapeutic strategy for glycogen storage diseases with accumulation of (aberrant) glycogen particles, including Pompe disease and adult polyglucosan body disease (APBD). The FDA-approved food additive guaiacol, which inhibits UDP-binding to GYS1, decreased GYS1 activity and glycogen content, and improved the APBD phenotype in a mouse model [60]. Nevertheless, the pleiotropic effects of guaiacol make it less suitable for targeted therapies. The safety and maximum tolerated dose of a novel GYS1-specific inhibitor, which potently inhibits human GYS1 in cell lysates of non-cancerous cells, is currently recruiting patients in a phase I clinical trial (NCT05249621) [61]. Inborn homozygous *GYS1* deficiency can lead to cardiomyopathy and cardiac arrest in some patients [36]. Nevertheless, side effects of drug-induced GYS1 inhibition might be tolerable for adults with normal cardiac function, as this inhibition is unlikely to be as complete as a complete *GYS1* deficiency. Our findings on the widespread presence of GYS1 in primary breast tumors and the involvement of GYS1 in proliferation make it of interest to extend development and evaluation of GYS1 inhibitors to breast cancer.

## Conclusions

The key glycogen synthesis enzyme GYS1 is expressed in most primary breast tumors, especially in triple-negative and Ki67-high tumors. Knockdown of *GYS1* impairs breast cancer cell proliferation and sensitizes to targeting of mitochondrial protein homeostasis. Our findings highlight GYS1 as potential therapeutic target in breast cancer, especially in TNBC and other highly proliferative subsets.

### Supplementary Information

**Additional file 1: Table S1.** Patient and tumor characteristics of included primary breast tumor samples.

**Additional file 2: Table S2.** Antibody and RNA specifications.

**Additional file 3: Figure S1.** METABRIC mRNA expression data (27) and patient overall survival. (a) Overall survival of breast cancer patients with high or low mRNA expression of the respective glycogen enzymes in their primary tumor. Curves are separated by the median mRNA expression. (b) Overall survival curves of triple-negative patients only. (c) Overall survival of respectively ER-/HER2+, ER+/HER2+ or ER+/HER2- breast cancer patients with high or low GYS1 mRNA expression in their primary tumor. Curves are separated by the median GYS1 expression. (d) Overall survival multivariate Cox regression analyses correcting for Nottingham Prognostic Index (NPI) for all (left) and triple-negative breast cancer patients (right) with respectively high and low GYS1 mRNA in their primary tumors. GBE1 = glycogen branching enzyme 1; GYS = glycogen synthase; PYGB = brain glycogen phosphorylase; PYGL = liver glycogen phosphorylase; PYGM = muscle glycogen phosphorylase

**Additional file 4: Figure S2.** METABRIC mRNA expression data and patient survival. (a) Spearman correlations among GYS1 mRNA and GYS isoform 2 (GYS2), glycogen branching enzyme 1 (GBE1), glycogen breakdown enzymes glycogen phosphorylase muscle isoform (PYGM), brain isoform (PYGB) and liver isoform (PYGL), the hypoxic marker carbonic anhydrase 9 (CA9), and Marker of Proliferation Ki67 (MKI67). Numbers indicate Spearman’s p, only significant correlations are displayed. (b) GYS1 mRNA log2 expression levels across breast cancer clinical subtypes (left panel), tumor stage (middle) and tumor grade (right). Boxes represent the interquartile range and median, whiskers 1.5*interquartile range. Only significant Wilcoxon P-values are annotated. *P<0.05, **P<0.01, ***P<0.005, ****P<0.001.

**Additional file 5: Figure S3.** Patient selection, survival curves and scoring examples of the primary breast tumor tissue micro-array. (a) Flowchart of the included tumor samples. (b) Corresponding PAS, PAS+diastase and CA9 staining of the areas depicted in Figure 1c. (c) Representative examples of staining intensity per scoring intensity and corresponding output of the analysis app in tumor cores and positive controls. (d) Additional boxplots of GYS1 and glycogen tumor H-score stratified by subtypes and Ki67 low vs. high subgroups within subtypes (top), and scores per tumor grade (bottom). (e) Heatmap of individual primary breast tumor samples (one sample per column) after unsupervised hierarchical clustering based on GYS1, glycogen and Ki67 tumor scores. White boxes in the annotation bars indicate missing values. (f) Overall survival curves of all patients included in the TMA analysis, stratified by respectively GYS1 or glycogen tertiles. Patients with more than one primary breast tumor (*n* = 9) were excluded from survival analyses.

**Additional file 6: Figure 4.** (a) Western blot of GYS1 demonstrates differential expression in breast cancer cell lines. (b) Knock-down of GYS1 in a broad panel of breast cancer cell lines, cultured in 5.6 mM glucose complete DMEM medium for 5 days, reduces cell growth in most breast cancer cell lines.

**Additional file 7: Figure 5.** (a) Western blot confirmation of siRNA GYS1 knockdown, belonging to Figure 2c.  (b) Clonogenic agarose assay of MDA-MB-231 cells with siCtrl or siGYS1, cultured in 5.6 mM glucose complete medium + 0.3% agarose for 14 days in normoxia. (c) Background intracellular glucose levels of MDA-MB-231 with scrambled control or GYS1 shRNA, after 48h culture in 5.6 mM glucose complete DMEM. (d) Proliferation of MDA-MB-231 cells with scrambled control or GYS1 shRNA, after 5 days culture in 10 mM complete DMEM.

**Additional file 8: Figure 6.** (a) Clonogenic agarose assay of HCC1806 cells transfected with siCtrl or siGYS1, cultured in 5.6 mM glucose complete medium for 10 days in normoxia. (b) Ki67 proliferation index in spheroids of HCC1806 cells transfected with siRNA-mediated GYS1 knockdown or scrambled control. (c) Well confluency of U87MG-shCtrl and -shGYS1 cells treated with different concentrations of GTPP, cultured in 5.6 mM glucose complete DMEM, was measured by Incucyte every 3h.

**Additional file 9: Figure 7.** Additional GYS1, PAS and CA9 xenograft stainings. (a) Whole-slide overview of GYS1 IHC in all xenograft tumors. Black arrows indicate GYS1-positive striated muscle cells. The 20x magnification of shGYS1 xenograft #7 shows re-expression of GYS1 in the bottom tumor half but not the upper part. (b) Xenograft qt-PCR GYS1 mRNA levels normalized to actin, confirming GYS1 knockdown in shGYS1 xenografts and the partial GYS1 regain in shGYS1 xenograft #7. (c) PAS staining +/- pretreatment with diastase and CA9 immunohistochemistry of xenograft tumor areas in Figure 3c. Magnification of GYS1 staining of the corresponding area in the shCtrl xenograft illustrates GYS1 expression was not restricted to CA9 positive areas (white arrows). Scalebars represent 1 mm for 1x magnification, 500 μm for 2x and 50 μm for 20x/40x. CA9 = carbonic anhydrase 9; GYS1 = glycogen synthase 1; PAS = periodic acid-Schiff.

## Data Availability

The METABRIC dataset used for mRNA analyses is publicly available at https://www.cbioportal.org/study/summary?id=brca_metabric (27). The other datasets used and/or analyzed during the current study are available from the corresponding author on reasonable request. Restrictions may apply for patient data due to national privacy laws.

## Abbreviations

APES: 3-Aminopropyltriethoxysilane
CA9: Carbonic anhydrase 9
CI: Confidence interval
ER: Estrogen receptor
FCS: Fetal calf serum
GBE1: Glycogen branching enzyme 1
GTPP: Gamitrinib-triphenylphosphnium
GYS: Glycogen synthase
HER2: Human epidermal growth factor receptor 2
HIF: Hypoxia-inducible factor
HR: Hazard ratio
IHC: Immunohistochemistry
MTT: 3-(4,5-Dimethylthiazol-2-yl)-2,5 diphenyltetrazolium methyl thiazolyl tetrazolium
PAS: Periodic acid-Schiff
PBS: Phosphate buffered saline
PVDF: Polyvinylidene fluoride
PYG: Glycogen phosphorylase
RMA: Robust multiarray average
STR: Short tandem repeat
TMA: Tissue-microarray
TNBC: Triple-negative breast cancer
UDP: Uridine diphosphate
UMCG: University Medical Center Groningen

## References

[1] Perou CM, Sørlie T, Eisen MB, van de Rijn M, Jeffrey SS, Rees CA (2000). Molecular portraits of human breast tumours. Nature.

[2] Bianchini G, Balko JM, Mayer IA, Sanders ME, Gianni L (2016). Triple-negative breast cancer: challenges and opportunities of a heterogeneous disease. Nat Rev Clin Oncol.

[3] Malorni L, Shetty PB, De Angelis C, Hilsenbeck S, Rimawi MF, Elledge R (2012). Clinical and biologic features of triple-negative breast cancers in a large cohort of patients with long-term follow-up. Breast Cancer Res Treat.

[4] de Heer EC, Jalving M, Harris AL (2020). HIFs, angiogenesis, and metabolism: elusive enemies in breast cancer. J Clin Invest.

[5] Network CGA (2012). Comprehensive molecular portraits of human breast tumours. Nature.

[6] Wigerup C, Pahlman S, Bexell D (2016). Therapeutic targeting of hypoxia and hypoxia-inducible factors in cancer. Pharmacol Ther.

[7] Zois CE, Harris AL (2016). Glycogen metabolism has a key role in the cancer microenvironment and provides new targets for cancer therapy. J Mol Med (Berl).

[8] Prats C, Graham TE, Shearer J (2018). The dynamic life of the glycogen granule. J Biol Chem.

[9] Roach PJ, Depaoli-Roach AA, Hurley TD, Tagliabracci VS (2012). Glycogen and its metabolism: some new developments and old themes. Biochem J.

[10] Zeqiraj E, Sicheri F (2015). Getting a handle on glycogen synthase - Its interaction with glycogenin. Mol Aspects Med.

[11] Ferrer JC, Favre C, Gomis RR, Fernández-Novell JM,García-Rocha M, de la Iglesia N,  (2003). Control of glycogen deposition. FEBS Lett.

[12] Pescador N, Villar D, Cifuentes D, Garcia-Rocha M, Ortiz-Barahona A, Vazquez S (2010). Hypoxia promotes glycogen accumulation through hypoxia inducible factor (HIF)-mediated induction of glycogen synthase 1. PLoS ONE.

[13] Favaro E, Bensaad K, Chong MG, Tennant DA, Ferguson DJ, Snell C (2012). Glucose utilization via glycogen phosphorylase sustains proliferation and prevents premature senescence in cancer cells. Cell Metab.

[14] Iida Y, Aoki K, Asakura T, Ueda K, Yanaihara N, Takakura S (2012). Hypoxia promotes glycogen synthesis and accumulation in human ovarian clear cell carcinoma. Int J Oncol.

[15] Ye IC, Fertig EJ, DiGiacomo JW, Considine M, Godet I, Gilkes DM (2018). Molecular Portrait of Hypoxia in Breast Cancer: A Prognostic Signature and Novel HIF-Regulated Genes. Mol Cancer Res.

[16] Rousset M, Zweibaum A, Fogh J (1981). Presence of glycogen and growth-related variations in 58 cultured human tumor cell lines of various tissue origins. Cancer Res.

[17] Shen GM, Zhang FL, Liu XL, Zhang JW (2010). Hypoxia-inducible factor 1-mediated regulation of PPP1R3C promotes glycogen accumulation in human MCF-7 cells under hypoxia. FEBS Lett.

[18] Pelletier J, Bellot G, Gounon P, Lacas-Gervais S, Pouyssegur J, Mazure NM (2012). Glycogen Synthesis is Induced in Hypoxia by the Hypoxia-Inducible Factor and Promotes Cancer Cell Survival. Front Oncol.

[19] Tang K, Zhu L, Chen J, Wang D, Zeng L, Chen C (2021). Hypoxia Promotes Breast Cancer Cell Growth by Activating a Glycogen Metabolic Program. Cancer Res.

[20] Matthews Q, Isabelle M, Harder SJ, Smazynski J, Beckham W, Brolo AG (2015). Radiation-Induced Glycogen Accumulation Detected by Single Cell Raman Spectroscopy Is Associated with Radioresistance that Can Be Reversed by Metformin. PLoS ONE.

[21] Meksiarun P, Aoki PHB, Van Nest SJ, Sobral-Filho RG, Lum JJ, Brolo AG (2018). Breast cancer subtype specific biochemical responses to radiation. Analyst.

[22] Curtis C, Shah SP, Chin SF, Turashvili G, Rueda OM, Dunning MJ (2012). The genomic and transcriptomic architecture of 2,000 breast tumours reveals novel subgroups. Nature.

[23] Wang P, Shan L, Xue L, Zheng B, Ying J, Lu N (2017). Genome wide copy number analyses of superficial esophageal squamous cell carcinoma with and without metastasis. Oncotarget.

[24] Lando M, Holden M, Bergersen LC, Svendsrud DH, Stokke T, Sundfør K (2009). Gene dosage, expression, and ontology analysis identifies driver genes in the carcinogenesis and chemoradioresistance of cervical cancer. PLoS Genet.

[25] Buart S, Terry S, Noman MZ, Lanoy E, Boutros C, Fogel P (2017). Transcriptional response to hypoxic stress in melanoma and prognostic potential of GBE1 and BNIP3. Oncotarget.

[26] Massari F, Ciccarese C, Santoni M, Iacovelli R, Mazzucchelli R, Piva F (2016). Metabolic phenotype of bladder cancer. Cancer Treat Rev.

[27] Ritterson Lew C, Guin S, Theodorescu D (2015). Targeting glycogen metabolism in bladder cancer. Nat Rev Urol.

[28] Nakamura-Tsuruta S, Yasuda M, Nakamura T, Shinoda E, Furuyashiki T, Kakutani R (2012). Comparative analysis of carbohydrate-binding specificities of two anti-glycogen monoclonal antibodies using ELISA and surface plasmon resonance. Carbohydr Res.

[29] Baba O (1993). Production of monoclonal antibody that recognizes glycogen and its application for immunohistochemistry. Kokubyo Gakkai Zasshi.

[30] Koopman T, Buikema HJ, Hollema H, de Bock GH, van der Vegt B (2018). Digital image analysis of Ki67 proliferation index in breast cancer using virtual dual staining on whole tissue sections: clinical validation and inter-platform agreement. Breast Cancer Res Treat.

[31] Cochrane EJ, Hulit J, Lagasse FP, Lechertier T, Stevenson B, Tudor C (2021). Impact of Mitochondrial Targeting Antibiotics on Mitochondrial Function and Proliferation of Cancer Cells. ACS Med Chem Lett.

[32] Broad Institute TCGA Genome Data Analysis Center. Analysis-ready standardized TCGA data from Broad GDAC Firehose 2016_01_28 run [Internet]. Broad Institute of MIT and Harvard. 2016.

[33] Haybittle JL, Blamey RW, Elston CW, Johnson J, Doyle PJ, Campbell FC (1982). A prognostic index in primary breast cancer. Br J Cancer.

[34] Cardoso F, Kyriakides S, Ohno S, Penault-Llorca F, Poortmans P, Rubio IT (2019). Early breast cancer: ESMO Clinical Practice Guidelines for diagnosis, treatment and follow-up. Ann Oncol.

[35] Kollberg G, Tulinius M, Gilljam T, Östman-Smith I, Forsander G, Jotorp P (2007). Cardiomyopathy and Exercise Intolerance in Muscle Glycogen Storage Disease 0. N Engl J Med.

[36] Cameron JM, Levandovskiy V, MacKay N, Utgikar R, Ackerley C, Chiasson D (2009). Identification of a novel mutation in GYS1 (muscle-specific glycogen synthase) resulting in sudden cardiac death, that is diagnosable from skin fibroblasts. Mol Genet Metab.

[37] Vranic S, Skenderi F, Beslagic V, Gataliza Z (2020). Glycogen-rich clear cell carcinoma of the breast: a comprehensive review. Appl Immunohistochem Mol Morphol.

[38] Kuroda H, Sakamoto G, Ohnisi K, Itoyama S (2005). Clinical and pathological features of glycogen-rich clear cell carcinoma of the breast. Breast Cancer.

[39] Georgescu TA, Munteanu O, Lisievici AC, Tebeică T, Crețoiu D, Toader O (2021). Glycogen-rich clear cell carcinoma of the breast with solid papillary pattern: Two cases with heterogeneous clinicopathological features. Exp Ther Med.

[40] Zhou Z, Kinslow CJ, Hibshoosh H, Guo H, Cheng SK, He C, et al. Clinical features, survival and prognostic factors of glycogen-rich clear cell carcinoma (GRCC) of the breast in the U.S. population. J Clin Med. 2019;8:246. 10.3390/jcm8020246 PMC6406344 30769905

[41] Tan PH, Ellis I, Allison K, Brogi E, Fox SB, Lakhani S (2020). The 2019 World Health Organization classification of tumours of the breast. Histopathology.

[42] Ibrahim MMH, Bheemanapally K, Sylvester PW, Briski KP (2020). Sex-specific estrogen regulation of hypothalamic astrocyte estrogen receptor expression and glycogen metabolism in rats. Mol Cell Endocrinol.

[43] Hochner-Celnikier D, Greenfield C, Finci-Yeheskel Z, Milwidsky A, Gutman A, Goldman-Wohl D (1997). Tamoxifen exerts oestrogen-agonistic effects on proliferation and plasminogen activation, but not on gelatinase activity, glycogen metabolism and p53 protein expression, in cultures of oestrogen-responsive human endometrial adenocarcinoma cells. Mol Hum Reprod.

[44] McCorvie TJ, Loria PM, Tu M, Han S, Shrestha L, Froese DS (2022). Molecular basis for the regulation of human glycogen synthase by phosphorylation and glucose-6-phosphate. Nat Struct Mol Biol.

[45] Altemus MA, Goo LE, Little AC, Yates JA, Cheriyan HG, Wu ZF (2019). Breast cancers utilize hypoxic glycogen stores via PYGB, the brain isoform of glycogen phosphorylase, to promote metastatic phenotypes. PLoS ONE.

[46] Shulman RG, Rothman DL (2017). The glycogen shunt maintains glycolytic homeostasis and the Warburg effect in cancer. Trends Cancer.

[47] Sun RC, Dukhande VV, Zhou Z, Young LEA, Emanuelle S, Brainson CF (2019). Nuclear Glycogenolysis Modulates Histone Acetylation in Human Non-Small Cell Lung Cancers. Cell Metab.

[48] Bhanot H, Reddy MM, Nonami A, Weisberg EL, Bonal D, Kirschmeier PT (2015). Pathological glycogenesis through glycogen synthase 1 and suppression of excessive AMP kinase activity in myeloid leukemia cells. Leukemia.

[49] Sun RC, Young LEA, Bruntz RC, Markussen KH, Zhou Z, Conroy LR (2021). Brain glycogen serves as a critical glucosamine cache required for protein glycosylation. Cell Metab.

[50] Dienel GA (2019). Does shuttling of glycogen-derived lactate from astrocytes to neurons take place during neurotransmission and memory consolidation?. J Neurosci Res.

[51] Xie H, Song J, Godfrey J, Riscal R, Skuli N, Nissim I (2021). Glycogen metabolism is dispensable for tumour progression in clear cell renal cell carcinoma. Nat Metab.

[52] Guin S, Pollard C, Ru Y, Ritterson Lew C, Duex JE, Dancik G, et al. Role in tumor growth of a glycogen debranching enzyme lost in glycogen storage disease. J Natl Cancer Inst. 2014;106:dju062. 10.1093/jnci/dju062 PMC4580555 24700805

[53] Nitschke F, Sullivan MA, Wang P, Zhao X, Chown EE, Perri AM (2017). Abnormal glycogen chain length pattern, not hyperphosphorylation, is critical in Lafora disease. EMBO Mol Med.

[54] Sullivan MA, Nitschke S, Skwara EP, Wang P, Zhao X, Pan XS (2019). Skeletal Muscle Glycogen Chain Length Correlates with Insolubility in Mouse Models of Polyglucosan-Associated Neurodegenerative Diseases. Cell Rep.

[55] Wang L, Wang M, Wise MJ, Liu Q, Yang T, Zhu Z (2020). Recent progress in the structure of glycogen serving as a durable energy reserve in bacteria. World J Microbiol Biotechnol.

[56] Henke BR, Sparks SM (2006). Glycogen phosphorylase inhibitors. Mini Rev Med Chem.

[57] Donnier-Maréchal M, Vidal S (2016). Glycogen phosphorylase inhibitors: a patent review (2013–2015). Expert Opin Ther Pat.

[58] Khan T, Sullivan MA, Gunter JH, Kryza T, Lyons N, He Y (2020). Revisiting Glycogen in Cancer: A Conspicuous and Targetable Enabler of Malignant Transformation. Front Oncol.

[59] Henke BR. Inhibition of glycogen phosphorylase as a strategy for the treatment of type 2 diabetes. In: Jones RM, editor. New therapeutic strategies for type 2 diabetes: small molecule approaches: The Royal Society of Chemistry. 2012. p. 324–65. ISBN: 1849735328.

[60] Kakhlon O, Ferreira I, Solmesky LJ, Khazanov N, Lossos A, Alvarez R (2018). Guaiacol as a drug candidate for treating adult polyglucosan body disease. JCI Insight.

[61] Tang B, Frasinyuk MS, Chikwana VM, Mahalingan KK, Morgan CA, Segvich DM (2020). Discovery and Development of Small-Molecule Inhibitors of Glycogen Synthase. J Med Chem.

