# Correction: Glycogen synthase 1 targeting reveals a metabolic vulnerability in triple-negative breast cancer

**DOI:** 10.1186/s13046-023-02800-3

**Published:** 2023-08-28

**Authors:** E. C. de Heer, C. E. Zois, E. Bridges, B. van der Vegt, H. Sheldon, W. A. Veldman, M. C. Zwager, T. van der Sluis, S. Haider, T. Morita, O. Baba, C. P. Schröder, S. de Jong, A. L. Harris, M. Jalving

**Affiliations:** 1 Department of Medical Oncology, University of Groningen, University Medical Center Groningen, PO Box 30.001, Groningen, 9700 RB The Netherlands; 2 Department of Oncology, Weatherall Institute of Molecular Medicine, Hypoxia and Angiogenesis Group, Cancer Research UK Molecular Oncology Laboratories, University of Oxford, Oxford, OX3 9DS UK; 3 Department of Radiotherapy and Oncology, School of Health, Democritus University of Thrace, Alexandroupolis, Greece; 4 Department of Oncology, MRC Weatherall Institute of Molecular Medicine, Molecular Oncology Laboratories, John Radclife Hospital, Oxford University, Oxford, OX3 9DS UK; 5 Department of Pathology and Medical Biology, University of Groningen, University Medical Center Groningen, Groningen, The Netherlands; 6 The Breast Cancer Now Toby Robins Research Centre, The Institute of Cancer Research, London, UK; 7 Tokushima University Graduate School, 3-18-15, Kuramoto-Cho, Tokushima, 770-8504 Japan; 8 Department of Medical Oncology, Antoni Van Leeuwenhoek-Netherlands Cancer Institute, Amsterdam, The Netherlands

**Correction: *J Exp Clin Cancer Res*42**, **143 (2023)**

10.1186/s13046-023-02715-z

Following publication of the original article [1], an error was identified in Additional File 8: Fig. 6c. HCC1806 should have been U87MG. The correct Additional File 8: Fig. 6c caption should be:

(c) Well confluency of U87MG-shCtrl and -shGYS1 cells treated with different concentrations of GTPP, cultured in 5.6 mM glucose complete DMEM, was measured by Incucyte every 3 h.

The correction does not affect the overall Conclusion of the article. The original article has been corrected.
