# Supplementary material for: Glycogen synthase 1 targeting reveals a metabolic vulnerability in triple-negative breast cancer
Source: J Exp Clin Cancer Res. 2023 Jun 6;42:143. doi: 10.1186/s13046-023-02715-z (PMC10242793; doi:10.1186/s13046-023-02715-z)
Supplement: Supplementary file 1 — Additional file 1: Table S1. Patient and tumor characteristics of included primary breast tumor samples. [file 13046_2023_2715_MOESM1_ESM.docx]

**Suppl. Table 1 Patient and tumor characteristics of included primary breast tumor samples**

| **Characteristic** | n = 337^1^ |
| --- | --- |
| **Receptor subtype** |  |
| ER+HER2- | 149 (44%) |
| ER+HER2+ | 68 (20%) |
| ER-HER2+ | 27 (8%) |
| Triple-negative | 93 (28%) |
| **Intrinsic subtype**  Luminal A  Luminal B  ER+, unspecified^2^  HER2  Basal-like | 123 (36%)  81 (24%)  13 (4%)  27 (8%)  93 (28%) |
| **Tumor side**  Left  Right  Bilateral | 150 (45%)  180 (53%)  7 (2%) |
| **Maximal tumor diameter (mm)** | 18 (12, 26) |
| **Pathological tumor size**  ≤2 cm  >2 cm & ≤5 cm  >5 cm  Unknown | 188 (56%)  130 (39%)  18 (5%)  1 (0%) |
| **Bloom Richardson grade**  Grade 1  Grade 2  Grade 3  Unknown | 49 (15%)  104 (31%)  183 (54%)  1 (0%) |
| **Pathological N-stage**  pN0  pN+ micrometastases  pN+ macrometastases  Unknown | 149 (44%)  60 (18%)  123 (36%)  5 (2%) |
| **Patient age at diagnosis (years)** | 54 (45, 63) |
| **Menopausal status** |  |
| Premenopausal | 94 (28%) |
| Perimenopausal | 37 (11%) |
| Postmenopausal | 157 (47%) |
| Unknown | 49 (15%) |
| **Received therapies**  Neoadjuvant chemotherapy  Surgery  Adjuvant radiotherapy  Adjuvant chemotherapy  Adjuvant hormonal therapy  Adjuvant HER2-targeted therapy | 8 (2%)  337 (100%)  223 (69%)  192 (57%)  164 (49%)  73 (22%) |
| **Follow-up (months)** | 135 (82, 153) |
| **Survival status**  Alive  Deceased  Unknown | 245 (73%)  83 (25%)  9 (3%) |
| ^1^Data are displayed as median (IQR) or n (%).  ^2^ i.e., ER+HER2- tumours with unevaluable Ki67 staining | |
