# Supplementary material for: Glycogen synthase 1 targeting reveals a metabolic vulnerability in triple-negative breast cancer
Source: J Exp Clin Cancer Res. 2023 Jun 6;42:143. doi: 10.1186/s13046-023-02715-z (PMC10242793; doi:10.1186/s13046-023-02715-z)
Supplement: Supplementary file 2 — Additional file 2: Table S2. Antibody and RNA specifications. [file 13046_2023_2715_MOESM2_ESM.docx]

**Suppl. Table 2 Antibody and RNA specifications**

| **Antibodies immunohistochemistry** | | | |
| --- | --- | --- | --- |
| *Antibody* | *Source* | *Origin* | *Dilution + antigen retrieval buffer* |
| GYS1 | Abcam ab40810 | Rabbit monoclonal | 1:200, Tris/HCl |
| Glycogen | Prof. O. Baba^ref37^ | Mouse monoclonal | 1:200, Tris/HCl |
| Ki67 | Roche Ventana 790-4286, clone 30-9 | Rabbit monoclonal | Ventana Ready-to-use Ultra Cell Conditioning Solution (Ultra CC1) |
| CA9 | Sigma SAB1300310 | Rabbit polyclonal | 1:200, Tris/EDTA |
| Cytokeratin 8/18 | Roche Cell Marque 760-4344, clone B22.1&B23.1 | Mouse monoclonal | Ventana Ready-to-use Ultra Cell Conditioning Solution (Ultra CC1) |
| **Antibodies Western Blot** | | | |
| *Antibody* | *Source* | *Origin* | *Dilution* |
| GYS1 | Cell Signaling Technology 3886S | Rabbit monoclonal | 1:1000 |
| pGYS1 | Abcam ab81230 | Rabbit monoclonal | 1:1000 |
| PYGL | HPA000962 | Rabbit polyclonal | 1:1000 |
| PYGB | HPA031067 | Rabbit polyclonal | 1:1000 |
| GBE1 | HPA038073 | Rabbit polyclonal | 1:1000 |
| CA9 | Sigma SAB1300310 | Rabbit polyclonal | 1:1000 |
| HIF1α | BD Biosciences 610959 | Mouse monoclonal | 1:1000 |
| Cleaved caspase 3 | Cell Signaling Technology 9661S | Rabbit polyclonal | 1:1000 |
| Cleaved PARP-1 | Cell Signaling Technology 5625 | Rabbit monoclonal | 1:1000 |
| B-actin | MP Biomedicals 69100 | Mouse monoclonal | 1:10000 |
| **RNA sequences** | | | |
| siRNAs  siCtrl – ON-target PLUS  siGYS1 - SmartPOOL | Dharmacon D001810-10-05  Invitrogen, sequences: 5’-GGGCGAGGAGCGUAACUAA-3’, 5’-CAACGACGCUGUCCUCUUU-3’, 5’-UAAGGAUUCAUAAAGCUUC-3’, 5’-GAAUCGGCCUCUUCAAUAG-3’ | | |
| shRNAs  shCtrl  shGYS1 | Sigma-Aldrich MISSION® short hairpin RNAs (shRNA)  SHC002V, sequence CCGGCAACAAGATGAAGAGCACCAACTCGAG  TTGGTGCTCTTCATCTTGTTGTTTTT  TRCN0000045693, sequence CCGGCCGCTATGAGTTCTCCAACAACTCGAG  TTGTTGGAGAACTCATAGCGGTTTTTG | | |
| Primers RNA sequencing  GYS1 sense  GYS1 anti-sense | 5’-CCGCTATGAGTTCTCCAACAAGG-3’  5’-AGAAGGCAACCACTGTCTGCTC-3’ | | |
