# Supplementary figures and images for: Glycogen synthase 1 targeting reveals a metabolic vulnerability in triple-negative breast cancer
Source: J Exp Clin Cancer Res. 2023 Jun 6;42:143. doi: 10.1186/s13046-023-02715-z (PMC10242793; doi:10.1186/s13046-023-02715-z)

# SUPPL FIGURE 1

a

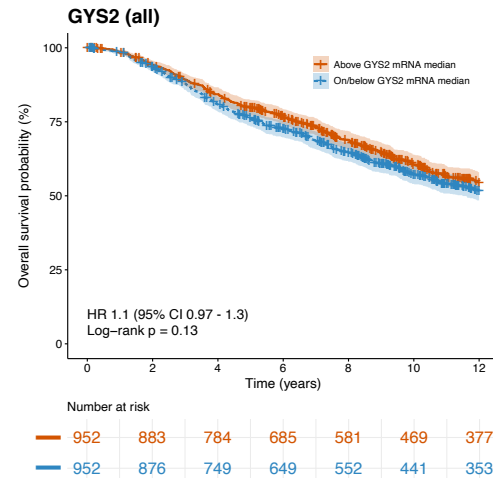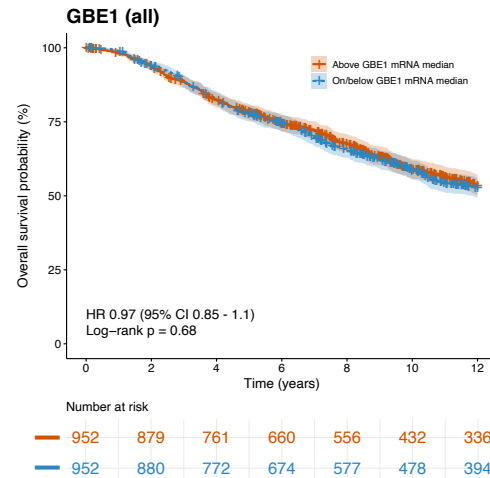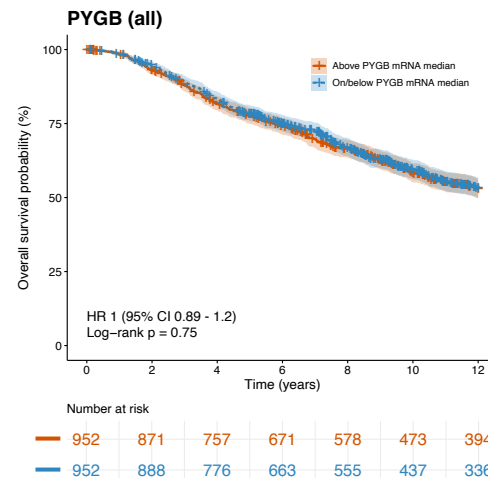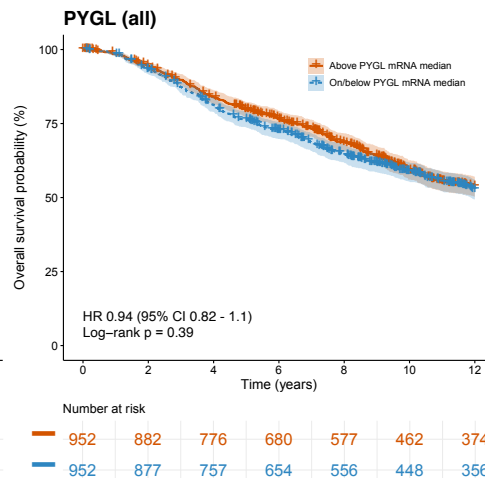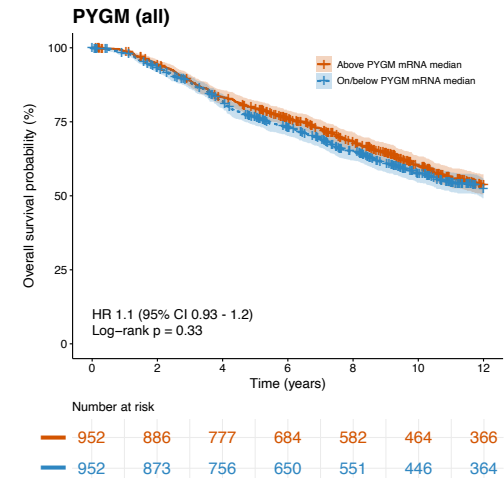

b

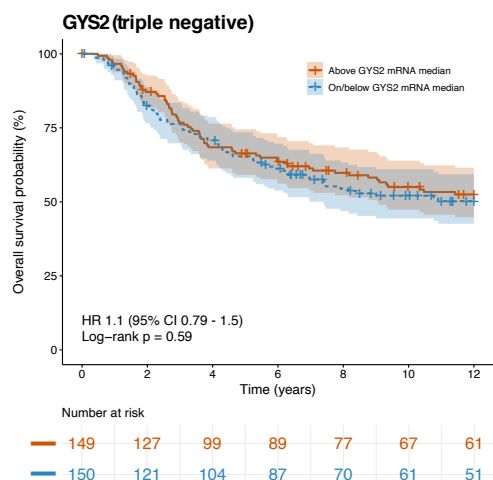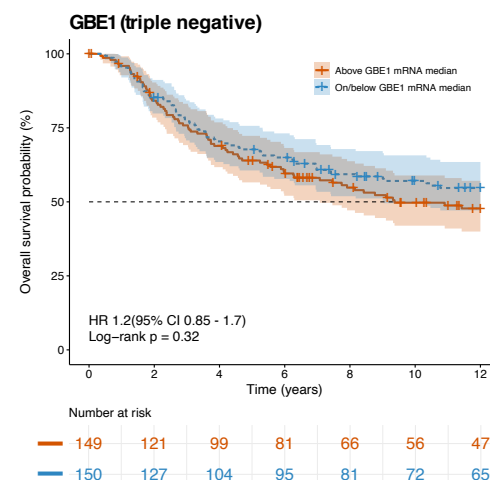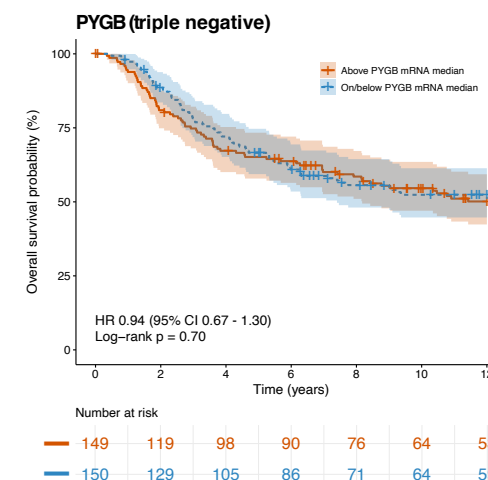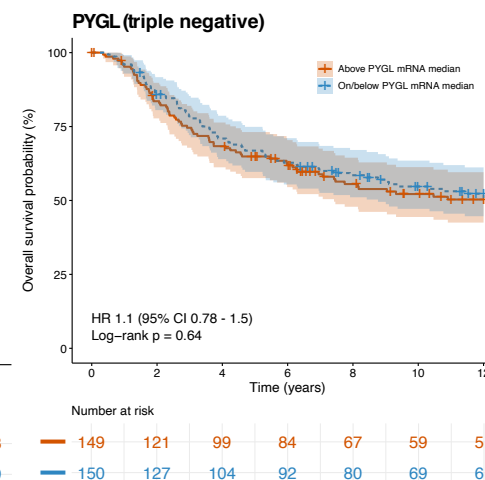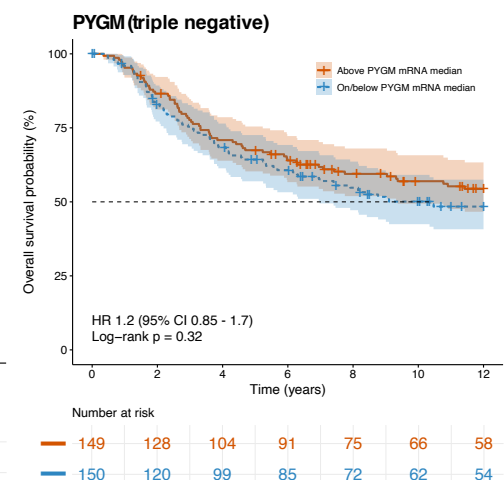

C

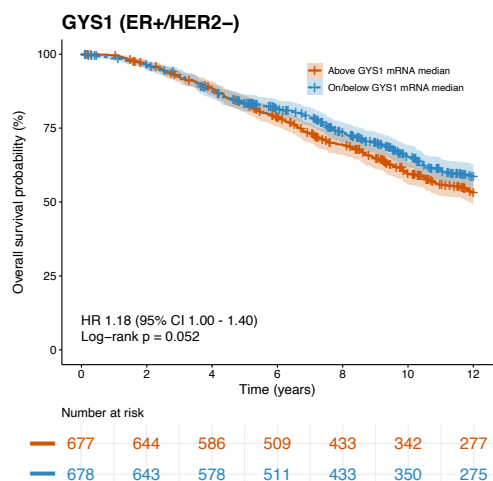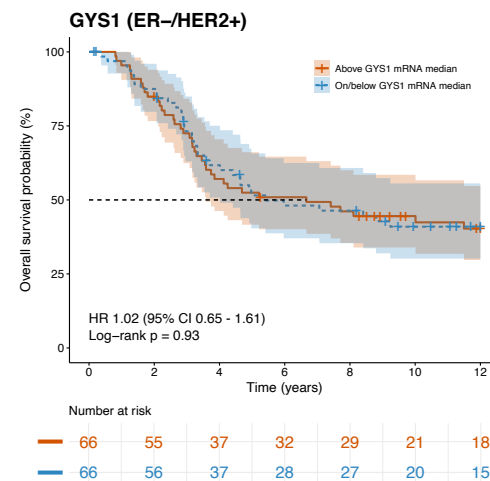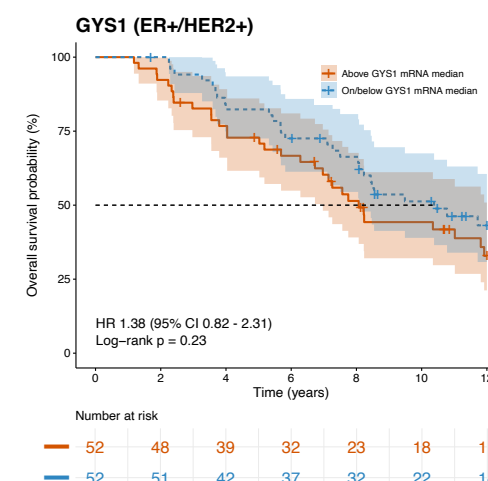

d

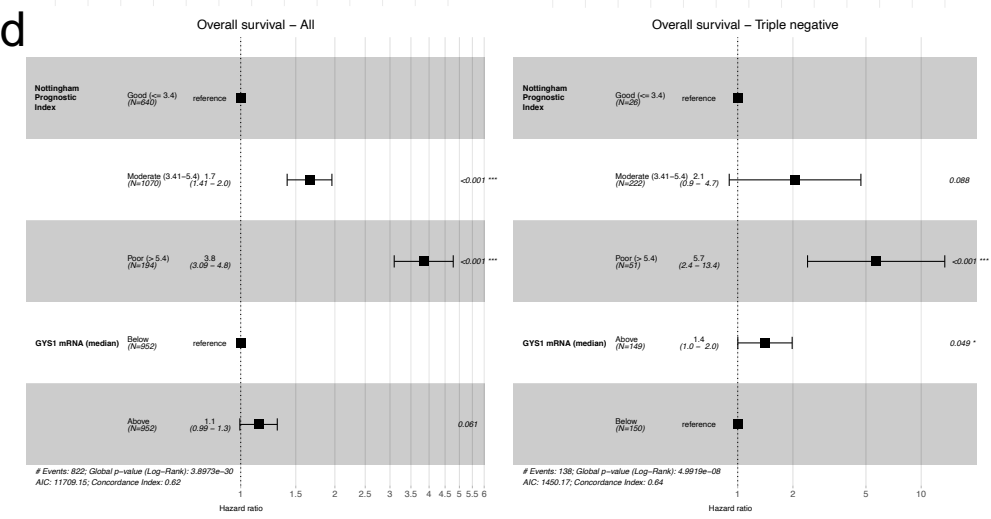

Supplement: Supplementary file 3 — Additional file 3: Figure S1. METABRIC mRNA expression data (27) and patient overall survival. (a) Overall survival of breast cancer patients with high or low mRNA expression of the respective glycogen enzymes in their primary tumor. Curves are separated by the median mRNA expression. (b) Overall survival curves of triple-negative patients only. (c) Overall survival of respectively ER-/HER2+, ER+/HER2+ or ER+/HER2- breast cancer patients with high or low GYS1 mRNA expression in their primary tumor. Curves are separated by the median GYS1 expression. (d) Overall survival multivariate Cox regression analyses correcting for Nottingham Prognostic Index (NPI) for all (left) and triple-negative breast cancer patients (right) with respectively high and low GYS1 mRNA in their primary tumors. GBE1 = glycogen branching enzyme 1; GYS = glycogen synthase; PYGB = brain glycogen phosphorylase; PYGL = liver glycogen phosphorylase; PYGM = muscle glycogen phosphorylase [file 13046_2023_2715_MOESM3_ESM.pdf]

# SUPPL. FIGURE 2

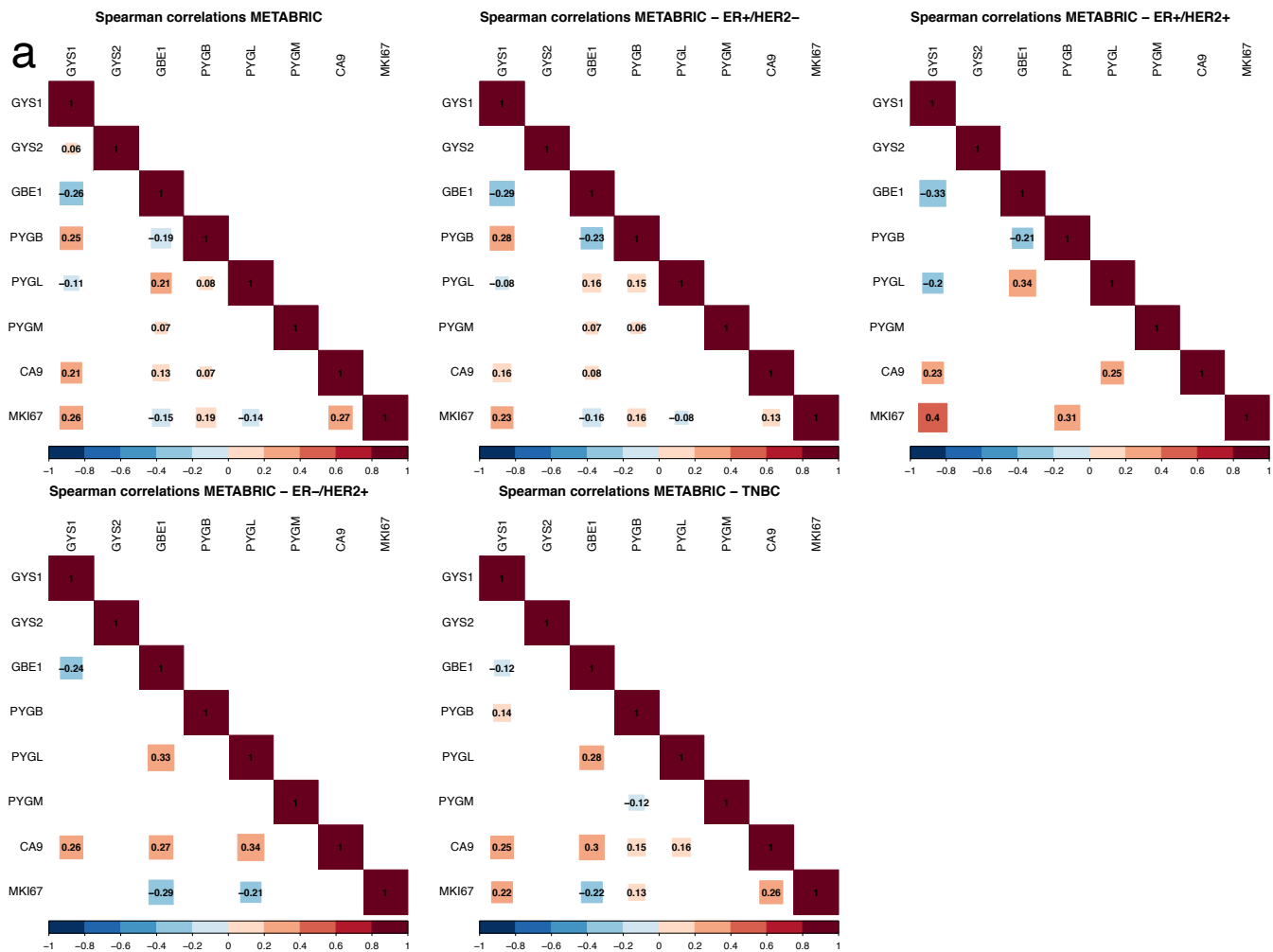

**b**

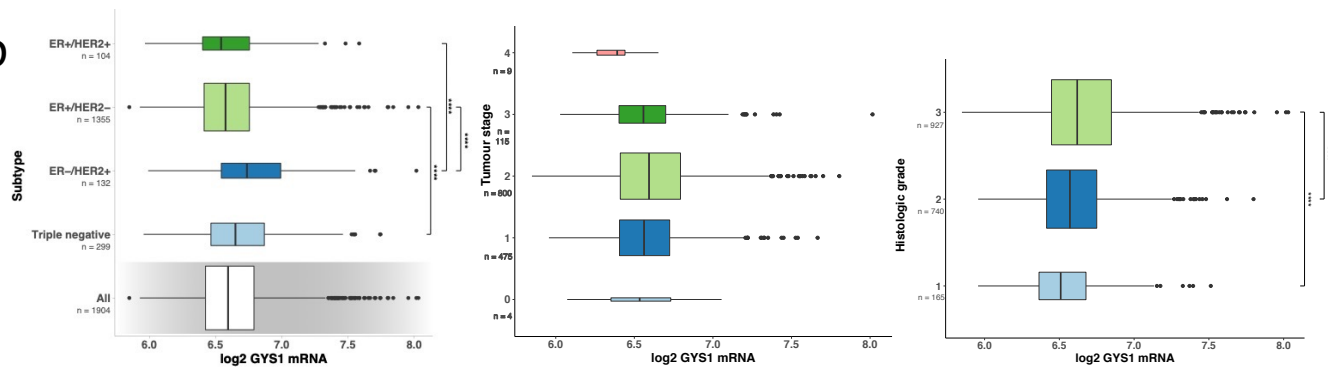

Supplement: Supplementary file 4 — Additional file 4: Figure S2. METABRIC mRNA expression data and patient survival. (a) Spearman correlations among GYS1 mRNA and GYS isoform 2 (GYS2), glycogen branching enzyme 1 (GBE1), glycogen breakdown enzymes glycogen phosphorylase muscle isoform (PYGM), brain isoform (PYGB) and liver isoform (PYGL), the hypoxic marker carbonic anhydrase 9 (CA9), and Marker of Proliferation Ki67 (MKI67). Numbers indicate Spearman’s p, only significant correlations are displayed. (b) GYS1 mRNA log2 expression levels across breast cancer clinical subtypes (left panel), tumor stage (middle) and tumor grade (right). Boxes represent the interquartile range and median, whiskers 1.5*interquartile range. Only significant Wilcoxon P-values are annotated. *P<0.05, **P<0.01, ***P<0.005, ****P<0.001. [file 13046_2023_2715_MOESM4_ESM.pdf]

SUPPL. FIGURE 3

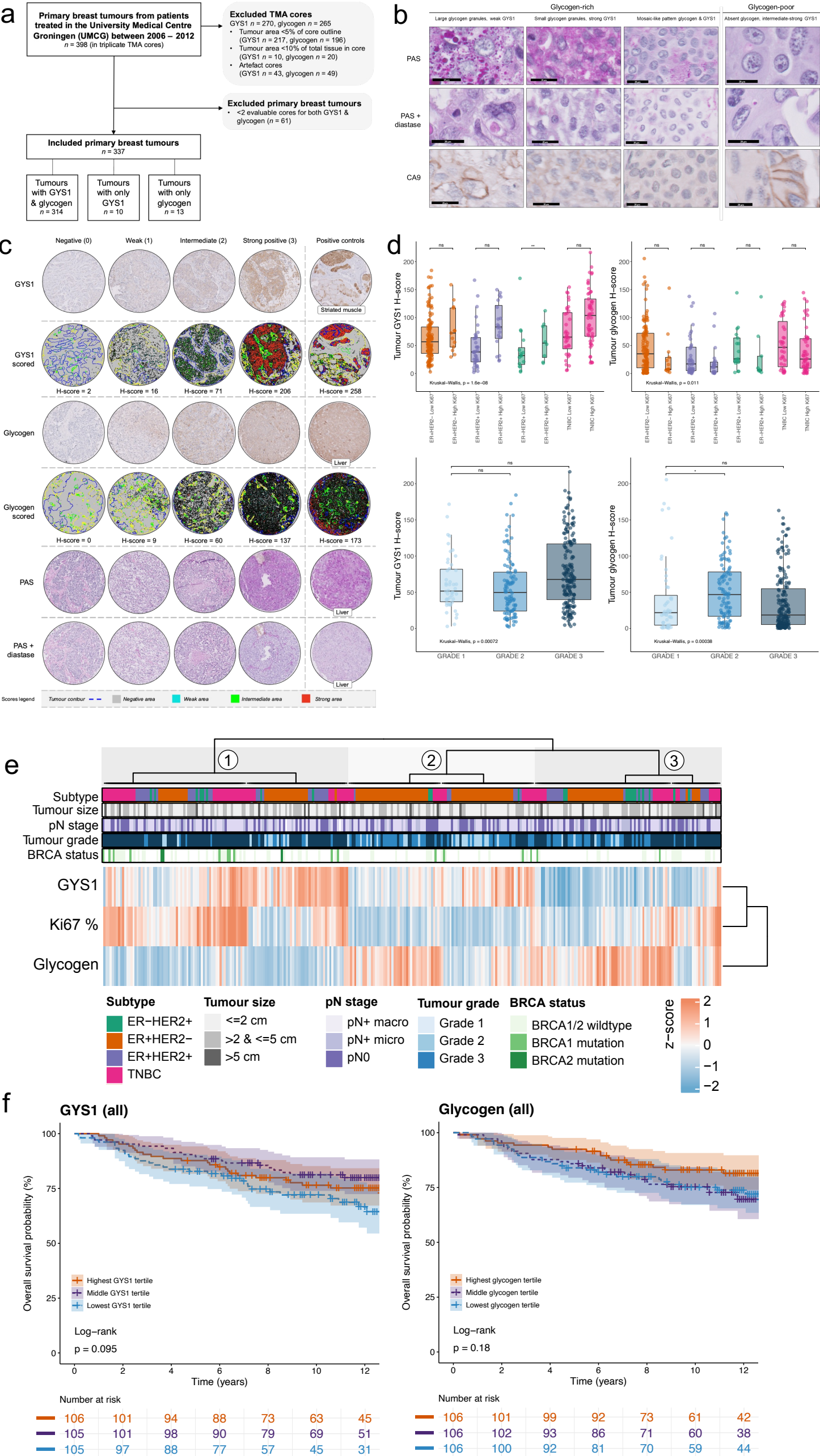

Supplement: Supplementary file 5 — Additional file 5: Figure S3. Patient selection, survival curves and scoring examples of the primary breast tumor tissue micro-array. (a) Flowchart of the included tumor samples. (b) Corresponding PAS, PAS+diastase and CA9 staining of the areas depicted in Figure 1c. (c) Representative examples of staining intensity per scoring intensity and corresponding output of the analysis app in tumor cores and positive controls. (d) Additional boxplots of GYS1 and glycogen tumor H-score stratified by subtypes and Ki67 low vs. high subgroups within subtypes (top), and scores per tumor grade (bottom). (e) Heatmap of individual primary breast tumor samples (one sample per column) after unsupervised hierarchical clustering based on GYS1, glycogen and Ki67 tumor scores. White boxes in the annotation bars indicate missing values. (f) Overall survival curves of all patients included in the TMA analysis, stratified by respectively GYS1 or glycogen tertiles. Patients with more than one primary breast tumor (n = 9) were excluded from survival analyses. [file 13046_2023_2715_MOESM5_ESM.pdf]

SUPPL. FIGURE 4

a

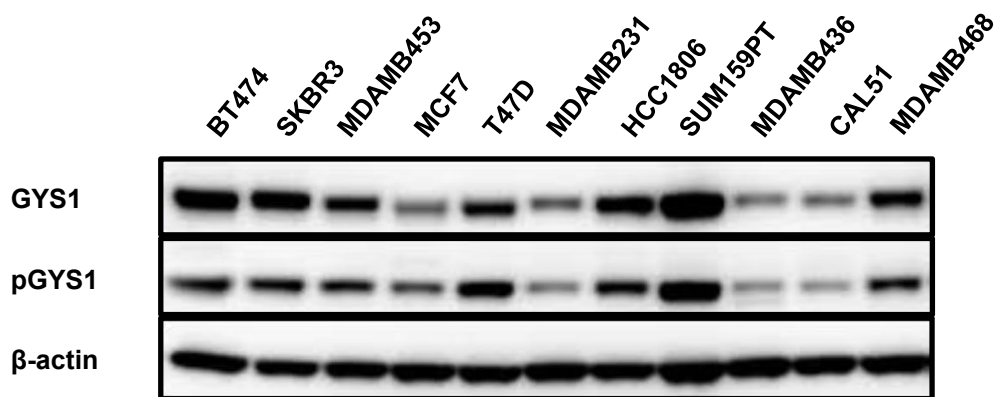

b

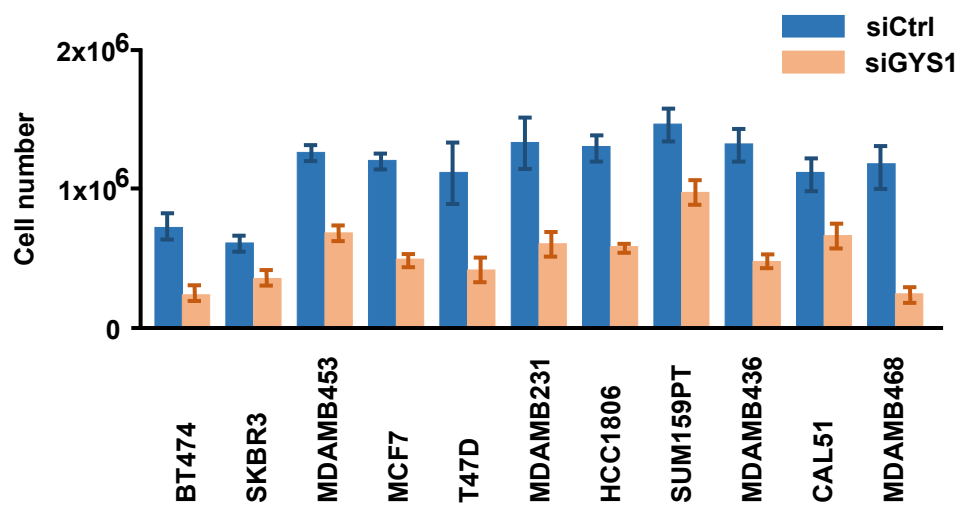

Supplement: Supplementary file 6 — Additional file 6: Figure 4. (a) Western blot of GYS1 demonstrates differential expression in breast cancer cell lines. (b) Knock-down of GYS1 in a broad panel of breast cancer cell lines, cultured in 5.6 mM glucose complete DMEM medium for 5 days, reduces cell growth in most breast cancer cell lines. [file 13046_2023_2715_MOESM6_ESM.pdf]

a

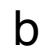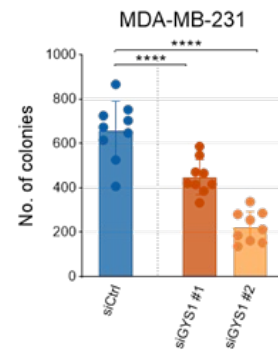

MDA-MB-231

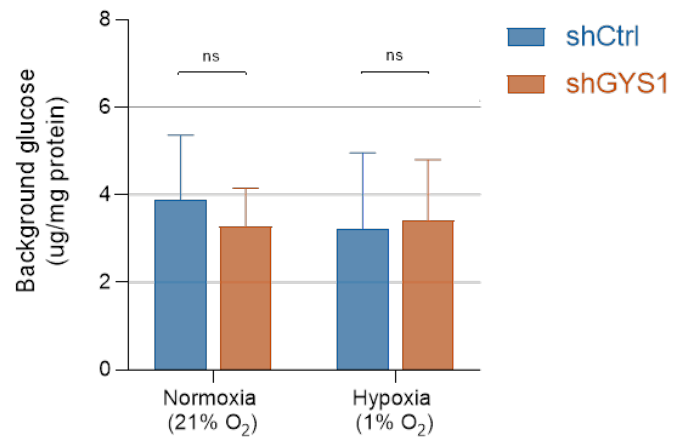

**d Cell proliferation (normoxia)**

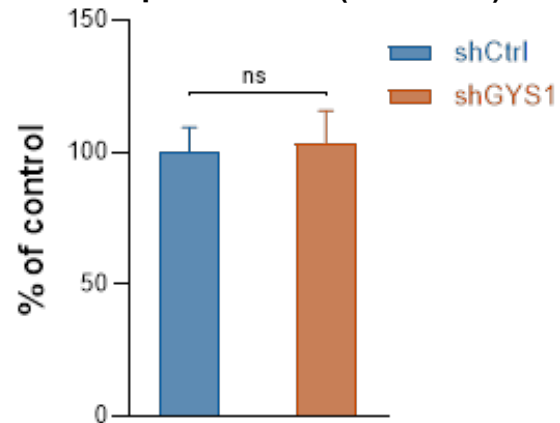

Supplement: Supplementary file 7 — Additional file 7: Figure 5. (a) Western blot confirmation of siRNA GYS1 knockdown, belonging to Figure 2c. (b) Clonogenic agarose assay of MDA-MB-231 cells with siCtrl or siGYS1, cultured in 5.6 mM glucose complete medium + 0.3% agarose for 14 days in normoxia. (c) Background intracellular glucose levels of MDA-MB-231 with scrambled control or GYS1 shRNA, after 48h culture in 5.6 mM glucose complete DMEM. (d) Proliferation of MDA-MB-231 cells with scrambled control or GYS1 shRNA, after 5 days culture in 10 mM complete DMEM. [file 13046_2023_2715_MOESM7_ESM.pdf]

SUPPL. FIGURE 6

a

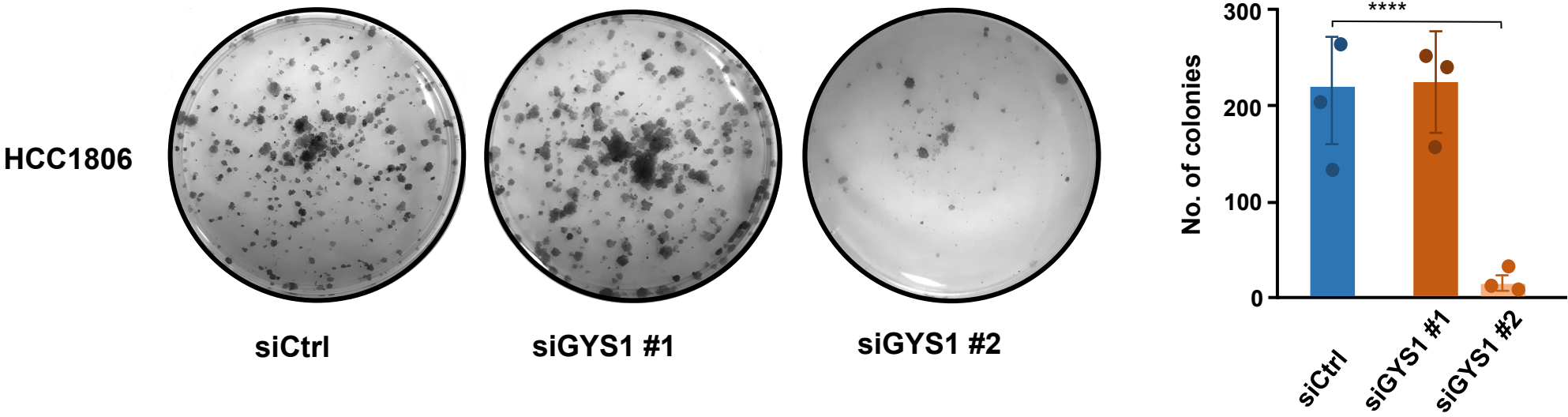

b

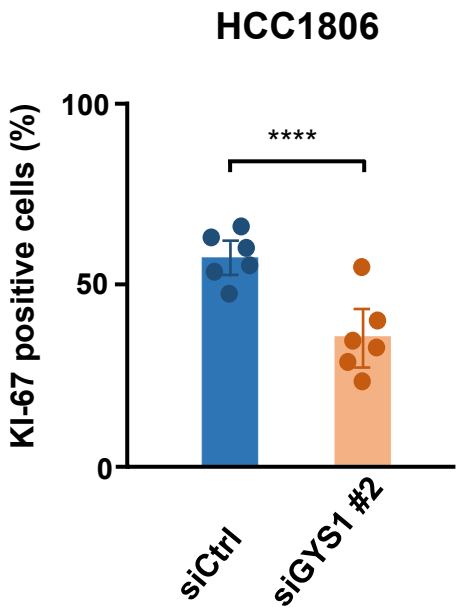

c

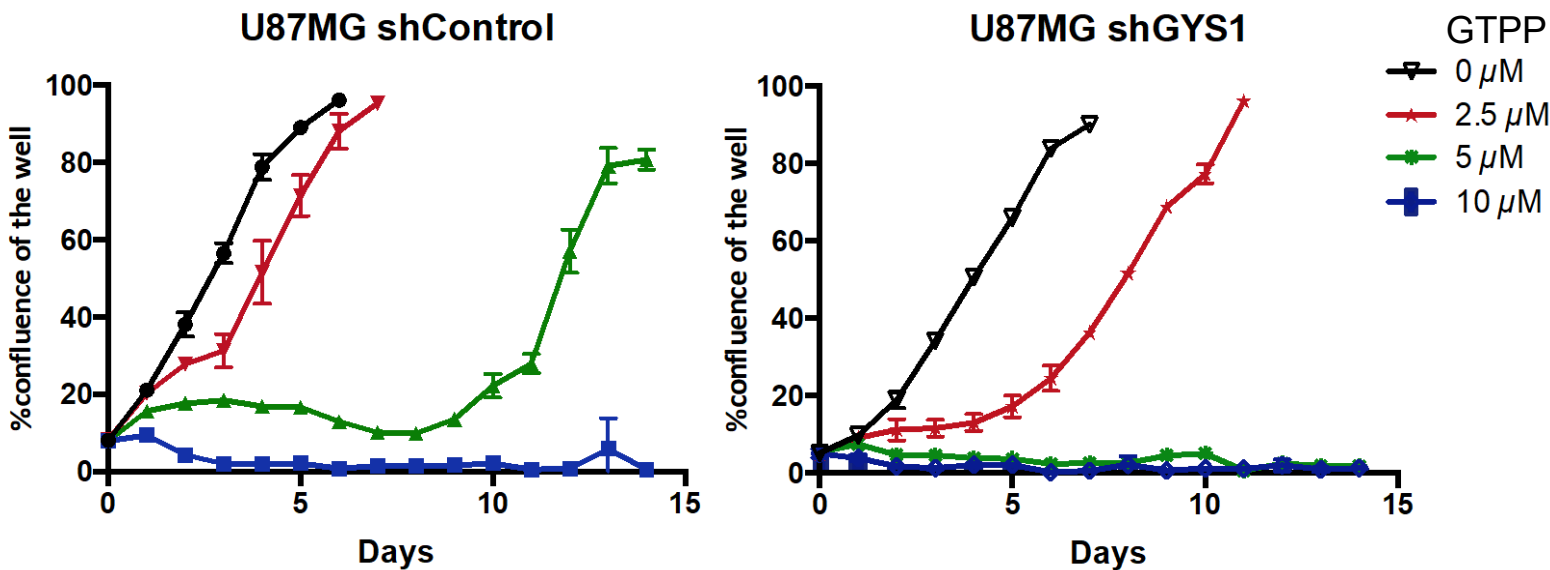

Supplement: Supplementary file 8 — Additional file 8: Figure 6. (a) Clonogenic agarose assay of HCC1806 cells transfected with siCtrl or siGYS1, cultured in 5.6 mM glucose complete medium for 10 days in normoxia. (b) Ki67 proliferation index in spheroids of HCC1806 cells transfected with siRNA-mediated GYS1 knockdown or scrambled control. (c) Well confluency of U87MG-shCtrl and -shGYS1 cells treated with different concentrations of GTPP, cultured in 5.6 mM glucose complete DMEM, was measured by Incucyte every 3h. [file 13046_2023_2715_MOESM8_ESM.pdf]
