# Supplementary material for: Glycogen synthase 1 targeting reveals a metabolic vulnerability in triple-negative breast cancer
Source: J Exp Clin Cancer Res. 2023 Jun 6;42:143. doi: 10.1186/s13046-023-02715-z (PMC10242793; doi:10.1186/s13046-023-02715-z)

SUPPL. FIGURE 7

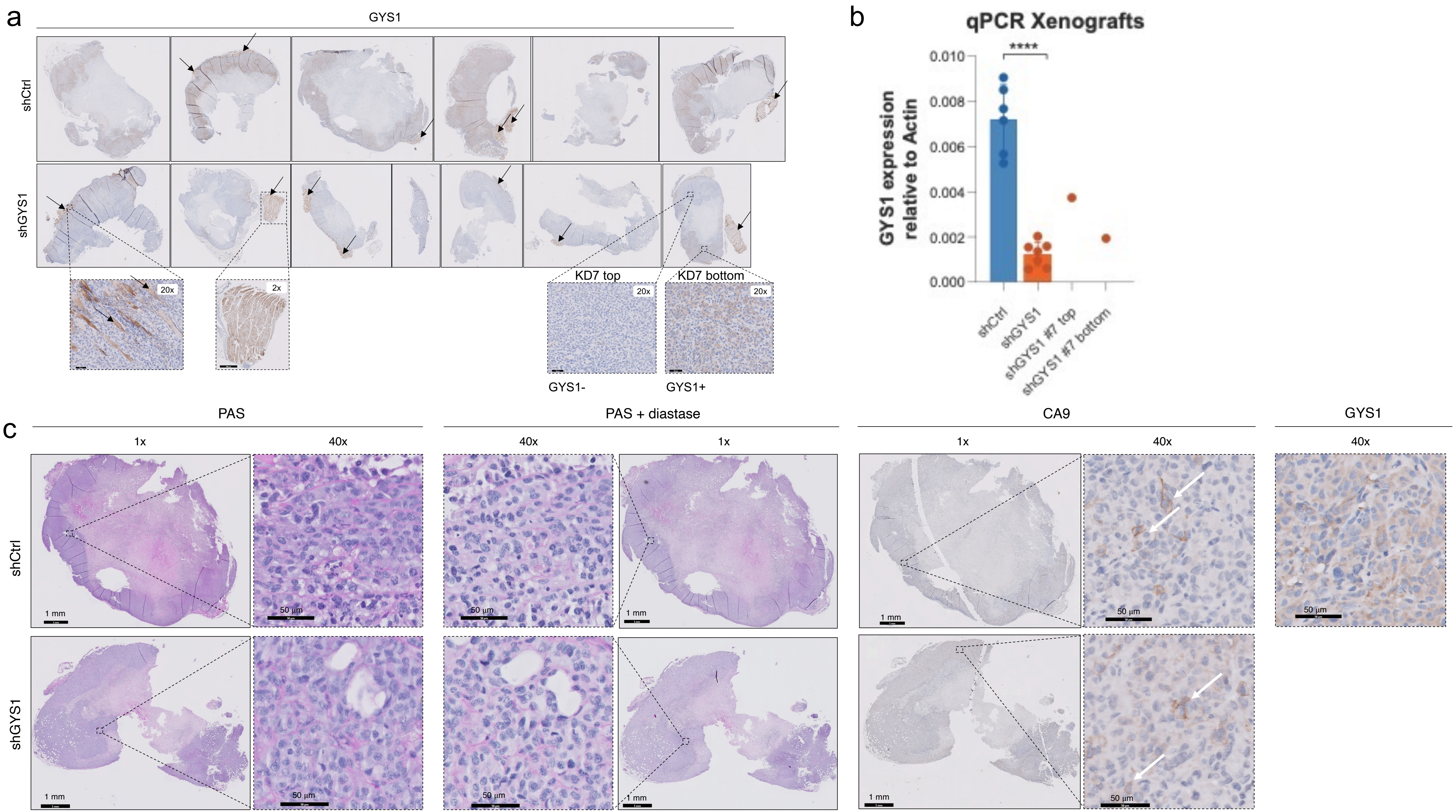

Supplement: Supplementary file 9 — Additional file 9: Figure 7. Additional GYS1, PAS and CA9 xenograft stainings. (a) Whole-slide overview of GYS1 IHC in all xenograft tumors. Black arrows indicate GYS1-positive striated muscle cells. The 20x magnification of shGYS1 xenograft #7 shows re-expression of GYS1 in the bottom tumor half but not the upper part. (b) Xenograft qt-PCR GYS1 mRNA levels normalized to actin, confirming GYS1 knockdown in shGYS1 xenografts and the partial GYS1 regain in shGYS1 xenograft #7. (c) PAS staining +/- pretreatment with diastase and CA9 immunohistochemistry of xenograft tumor areas in Figure 3c. Magnification of GYS1 staining of the corresponding area in the shCtrl xenograft illustrates GYS1 expression was not restricted to CA9 positive areas (white arrows). Scalebars represent 1 mm for 1x magnification, 500 μm for 2x and 50 μm for 20x/40x. CA9 = carbonic anhydrase 9; GYS1 = glycogen synthase 1; PAS = periodic acid-Schiff. [file 13046_2023_2715_MOESM9_ESM.pdf]
